# Identification and Characterization of Two Novel RNA Viruses from *Anopheles gambiae* Species Complex Mosquitoes

**DOI:** 10.1371/journal.pone.0153881

**Published:** 2016-05-03

**Authors:** Guillaume Carissimo, Karin Eiglmeier, Julie Reveillaud, Inge Holm, Mawlouth Diallo, Diawo Diallo, Amélie Vantaux, Saorin Kim, Didier Ménard, Sovannaroth Siv, Eugeni Belda, Emmanuel Bischoff, Christophe Antoniewski, Kenneth D. Vernick

**Affiliations:** 1 Institut Pasteur, Unit of Insect Vector Genetics and Genomics, Department of Parasites and Insect Vectors, 28 rue du Docteur Roux, Paris, 75015, France; 2 CNRS, Unit of Hosts, Vectors and Pathogens (URA3012), 28 rue du Docteur Roux, Paris, 75015, France; 3 Laboratory of Microbial Immunity, Singapore Immunology Network, Agency for Science, Technology and Research (A(*)STAR), Singapore, Singapore; 4 INRA, UMR 1309 CMAEE, Montpellier, France; 5 Institut Pasteur de Dakar, Dakar, Senegal; 6 Institut Pasteur of Cambodia, Phnom Penh, Cambodia; 7 National Center for Parasitology Entomology and Malaria Control, Phnom Penh, Cambodia; 8 Sorbonne Universités, UPMC University of Paris 06, CNRS UMR 7622 Institut de Biologie Paris Seine, Drosophila Genetics and Epigenetics, F-75005, Paris, France; 9 Sorbonne Universités, UPMC University of Paris 06, CNRS FR3631 Institut de Biologie Paris Seine, ARTbio Bioinformatics Analysis Facility, F-75005, Paris, France; 10 Department of Microbiology, University of Minnesota, Minneapolis, Minnesota, 55108, United States of America; University of British Columbia, CANADA

## Abstract

Mosquitoes of the *Anopheles gambiae* complex display strong preference for human bloodmeals and are major malaria vectors in Africa. However, their interaction with viruses or role in arbovirus transmission during epidemics has been little examined, with the exception of O’nyong-nyong virus, closely related to Chikungunya virus. Deep-sequencing has revealed different RNA viruses in natural insect viromes, but none have been previously described in the *Anopheles gambiae* species complex. Here, we describe two novel insect RNA viruses, a Dicistrovirus and a Cypovirus, found in laboratory colonies of *An*. *gambiae* taxa using small-RNA deep sequencing. Sequence analysis was done with Metavisitor, an open-source bioinformatic pipeline for virus discovery and *de novo* genome assembly. Wild-collected *Anopheles* from Senegal and Cambodia were positive for the Dicistrovirus and Cypovirus, displaying high sequence identity to the laboratory-derived virus. Thus, the Dicistrovirus (*Anopheles* C virus, AnCV) and Cypovirus (*Anopheles* Cypovirus, AnCPV) are components of the natural virome of at least some anopheline species. Their possible influence on mosquito immunity or transmission of other pathogens is unknown. These natural viruses could be developed as models for the study of *Anopheles*-RNA virus interactions in low security laboratory settings, in an analogous manner to the use of rodent malaria parasites for studies of mosquito anti-parasite immunity.

## Introduction

Insect specific RNA viruses from various families (i.e. *Flaviviridae*, *Togaviridae*, *Bunyaviridae*, *Rhabdoviridae*, *Mesoniviridae*) and the taxon Negevirus have been described in *Anopheles* mosquitoes [1–5]. These viruses have been discovered by isolation from cell cultures, by RT-PCR and manual sequencing targeting regions of known viruses, or using deep sequencing on field caught insect samples [6, 7]. The siRNA pathway of mosquitoes is involved in the interaction and processing of the viral double strand RNA (dsRNA) intermediates produced by RNA viruses [8–14]. Deep sequencing of small RNA and bioinformatics has been used to reconstruct active novel viruses in plants, Drosophila or mosquitoes, using detection of viral-derived small interfering RNAs (viRNAs) as a criterion for active replication [7, 15, 16]. Small RNA deep sequencing should allow sensitive detection and discovery of viruses that produce dsRNA intermediates. Alternately, viral discovery by inoculation of extracts onto cell lines is not limited to detection of dsRNA intermediates, but can be biased due to differential efficiency of viral replication across cell lineages [17–19].

Among *Anopheles* mosquitoes, the most mature reference genome sequence and genomic tools have been developed for the *An*. *gambiae* complex, particularly the sister taxa *An*. *gambiae* and *An*. *coluzzii*, and these are currently the most powerful *Anopheles* models. Within the African malaria vectors of the *An*. *gambiae* complex, to our knowledge no natural RNA viruses have yet been described, with the exception of O’nyong-nyong virus (ONNV), a pathogenic arbovirus transmitted to humans [20], which is a close relative of Chikungunya virus transmitted by *Aedes* mosquitoes [21, 22]. Consequently, research on viral response and antiviral immunity of *Anopheles* has been limited to ONNV infections [14, 23–27], but research using ONNV requires sophisticated biosecurity conditions. Identification of *Anopheles* RNA viruses that could be used as low-biosecurity model systems would facilitate studies of *Anopheles* antiviral immunity and mosquito-virus interactions.

In the current study, we used small RNA deep-sequence datasets to reconstruct more than 90% of a novel Dicistrovirus, and several segments of a previously unknown Cypovirus. We verified their infection prevalence within the *Anopheles* colony where they were discovered. Oral infection experiments were used to evaluate the species-specificity of these newly discovered viral strains and their ability to be transmitted to the progeny. Finally, we detected both viruses in wild *Anopheles* mosquitoes captured in Cambodia and in Senegal, confirming that they are components of the natural virome.

The results indicate that a laboratory *Anopheles* colony, widely used to study vector interactions with different pathogens, carries previously undetected natural insect viral infections that could potentially influence the results of such studies. These novel insect viruses also could become valuable tools to study *Anopheles* antiviral immunity, as well as the underlying mechanisms of vector interactions with RNA viruses, without the requirement for high biosecurity infrastructure or procedures.

## Results

Four datasets of small RNA sequence generated from pooled *An*. *coluzzii* (Ngousso colony) mosquito midguts were analyzed using the Metavisitor pipeline [28], a suite of open source bioinformatic tools designed to simplify virus diagnostic, discovery and genome reconstruction on a Galaxy framework [29]. Two novel viruses were detected in the mosquitoes, a Cypovirus (genus: *Cypovirus*, sub-family: *Spinareovirinae*, family: *Reoviridae*), and a Dicistrovirus (genus: *Cripavirus*, family: *Dicistroviridae*, order: *Picornavirales*).

### Detection of a Cypovirus

We detected a transcript (accession number KU169880) of 892 nt aligning to segment 10 of *Culex restuans* Cypovirus (CrCPV)[30] and to *Uranotaenia sapphirina* Cypovirus (UsCPV)[31] with 91% and 83% identity, respectively. Multiple alignments of the nucleic sequences with Clustal O [32] indicated that it was not a chimeric segment of the two known viruses. This segment possesses an intact 237 aa ORF that codes for a complete polyhedrin protein with 96% and 90% amino acid identity to the polyhedrin of CrCPV and UsCPV, respectively. Cypovirus genomes are comprised of 10 to 11 segments of double strand RNA, each coding for a different viral protein. The polyhedrin, protein, coded by segment 10, is highly conserved among Cypoviruses.

**Fig 1:**
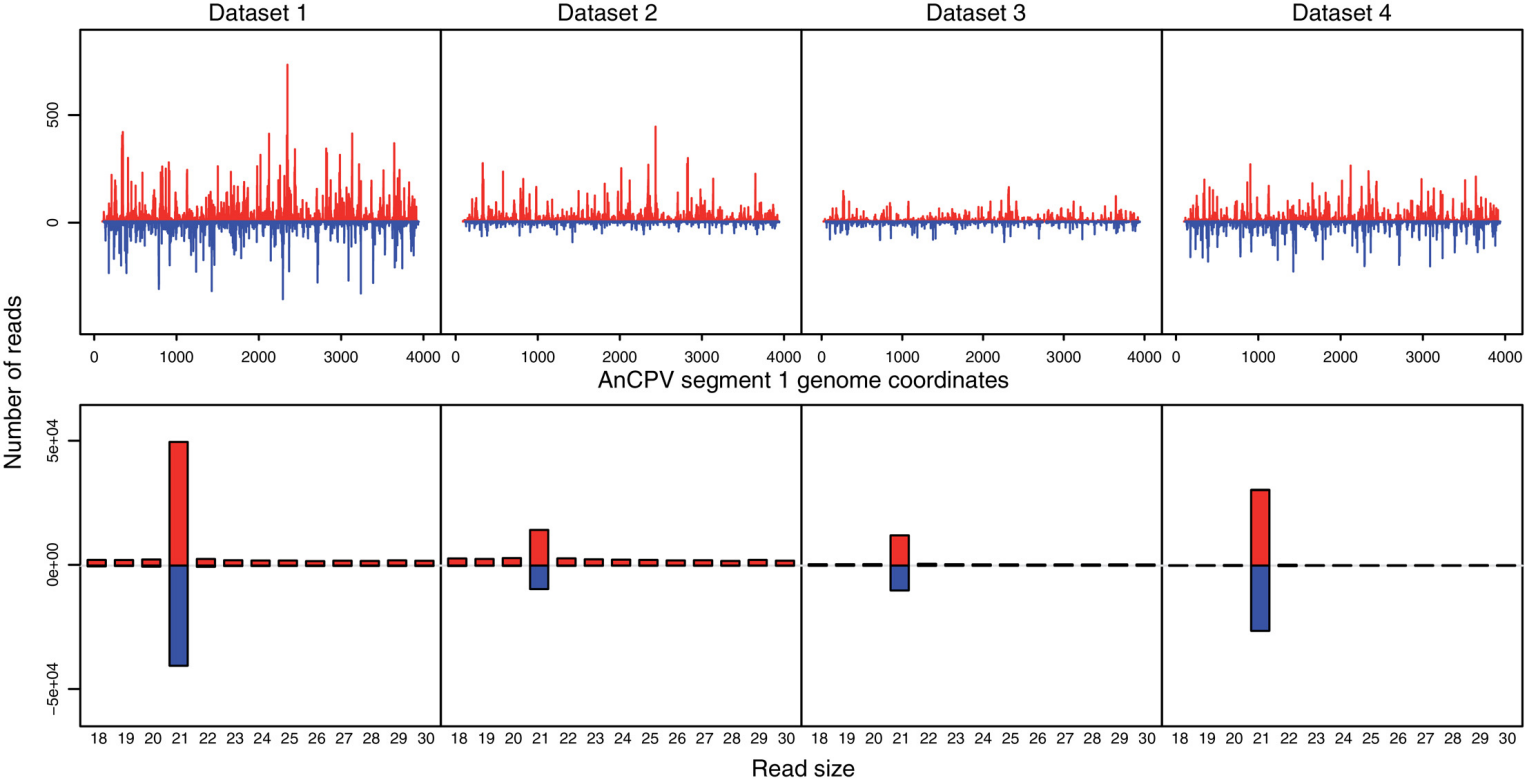
Readmaps and size distributions of small RNAs that align on the *Anopheles* Cypovirus reconstructed RdRp genomic sequence. The four small RNA datasets were independently aligned to the sequence of *Anopheles* Cypovirus segment 1 (accession number KU169879) with bowtie allowing no mismatches. From the bowtie output, lattice maps and read size histograms were generated representing the positions and size of the small RNAs that align to the viral genomic segment.

A second contig of 3889 nt was reconstructed (accession number KU169879) containing an ORF coding for a 1246 aa protein that has 34% identity with the RNA dependent RNA polymerase (RdRp) of *Bombyx mori* Cypovirus 1 (ACX54961.1) over 98% of its sequence. Realignment of the original small RNA reads to the contig shows a dicing profile with the read length peaking at 21nt in all four datasets (Fig 1). This is the characteristic product of viral replication intermediates processed by the siRNA pathway [33], indicating that the reconstructed sequences belong to an actively replicating virus infecting the mosquitoes.

**Fig 2:**
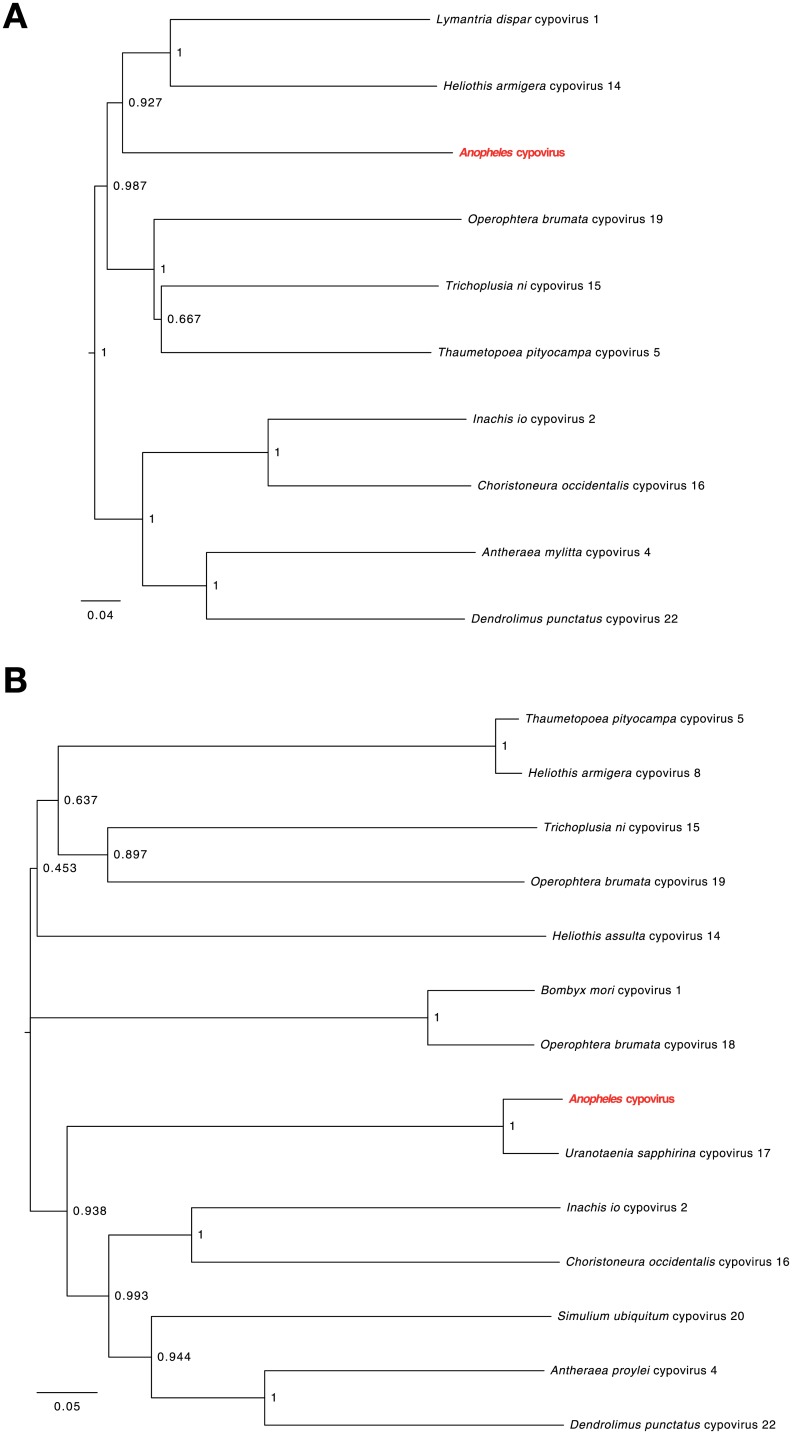
AnCPV RNA dependent RNA polymerase and polyhedrin phylogeny. (A) Neighbor-joining tree of RNA-dependent RNA polymerase (RdRp) sequences of Cypoviruses, (B) Neighbor-joining tree of polyhedrin sequences of Cypoviruses. Multiple protein alignment was performed with the longest ORF (corresponding to the polymerase) for each nucleic acid. NJ bootstrap values (above 0.75) are indicated above branches. Pairwise distances are indicated in S6 File.

Phylogenetic analysis of the predicted polymerase and polyhedrin peptide sequences were performed using the available sequences for the different *Cypovirus* species (Fig 2). Neighbor-joining trees show that reconstructed RdRp and polyhedrin sequences are related but distinct from known Cypoviruses. The CPVs have been classified into 16 types by the International Committee on Taxonomy of Viruses [34], or 21 types based on the electrophoretic migration patterns of their dsRNA genome segments [35]. Therefore, it is not surprising that the phylogeny of these viruses inferred from the polymerase sequence, and to a lesser extent from the polyhedrin gene, does not reflect the virus types classification. We conclude that the reconstructed sequences are evidence of a previously unknown virus, here named *Anopheles* Cypovirus (AnCPV). We hypothesize that AnCPV is a segmented (10 to 11 segments) double stranded RNA virus of the genus *Cypovirus*, sub-family *Spinareovirinae*, family *Reoviridae*. Based on the phylogenetic tree on the polyhedrin gene, this virus could be a novel member of the Cypovirus type 17 [35].

**Fig 3:**
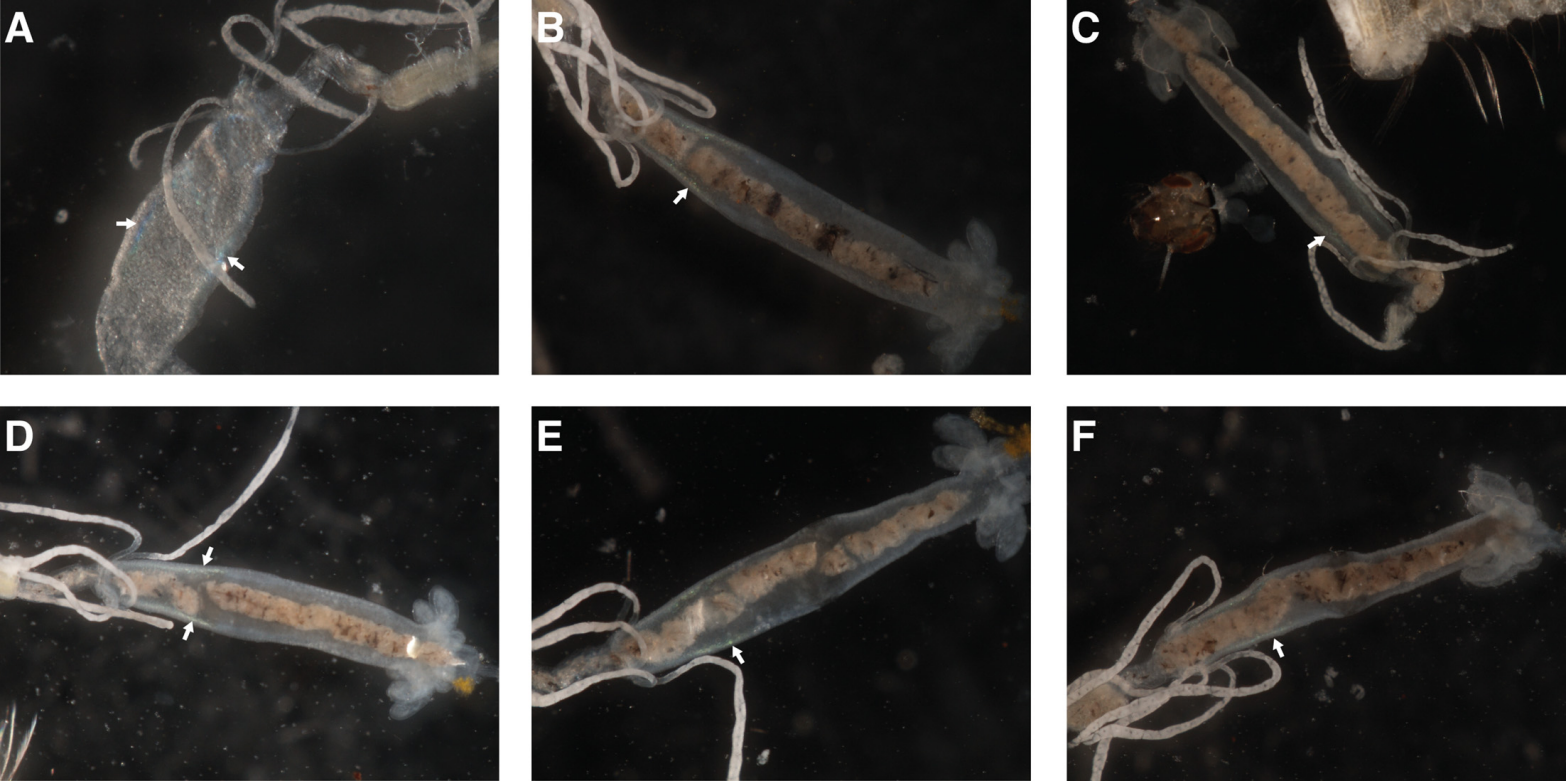
Microscopic observation of *Anopheles coluzzii* larvae midguts. Signs of Cypoviral infection are white-blue iridescent areas as indicated by the white arrows in each panel. Each panel corresponds to a different mosquito larva.

Light microscopy of *An*. *coluzzii* larval midguts displayed gross pathological signs of Cypovirus infection, detectable as localized clusters of tightly packed inclusion bodies in the midgut epithelium that appear visually as iridescent blue-white features [31] (white arrows, Fig 3). RT-PCR primers were designed based on the sequence of segments 1 and 10 and successfully used to amplify fragments from viral RNA templates in the total RNA used for the original small RNA library preparation. Sanger sequencing of the amplicons showed no variation from the bioinformatically reconstructed genomic sequences (S1 Zipped Archive).

### Detection of a Dicistrovirus

Other contigs reconstructed from the same small RNA datasets displayed significant similarity using blastx to *Drosophila* C virus (DCV) and Cricket paralysis virus (CrPV) from the *Distroviridae* family. Using additional long paired-end RNAseq datasets (accession number ERS977505), we were able to reconstruct a 8919 nt viral genomic sequence that completely includes these contigs and contains two ORFs (accession number KU169878). The first ORF (5’ ORF) codes for an 1802 aa (nucleotides 391 to 5799 in the reconstructed genome) polyprotein with 48% blastp identity to DCV nonstructural polyprotein. The second ORF (3’ ORF) codes for a predicted 852 aa protein (nucleotides 6067 to 8625 on the reconstructed genome) and displays 54% blastp identity to DCV structural polyprotein. This dicistronic organization of the reconstructed genome is typical of *Dicistroviridae* with an ORF1 that encodes for non-structural proteins and an ORF2 that encodes for virion proteins (reviewed in [36]). *Dicistroviridae* are related to the *Picornaviridae*, which encode the same proteins in a monocistronic manner as one polyprotein, and to the *Comoviridae*, which encode the two ORFs but on two monocistronic RNAs.

**Fig 4:**
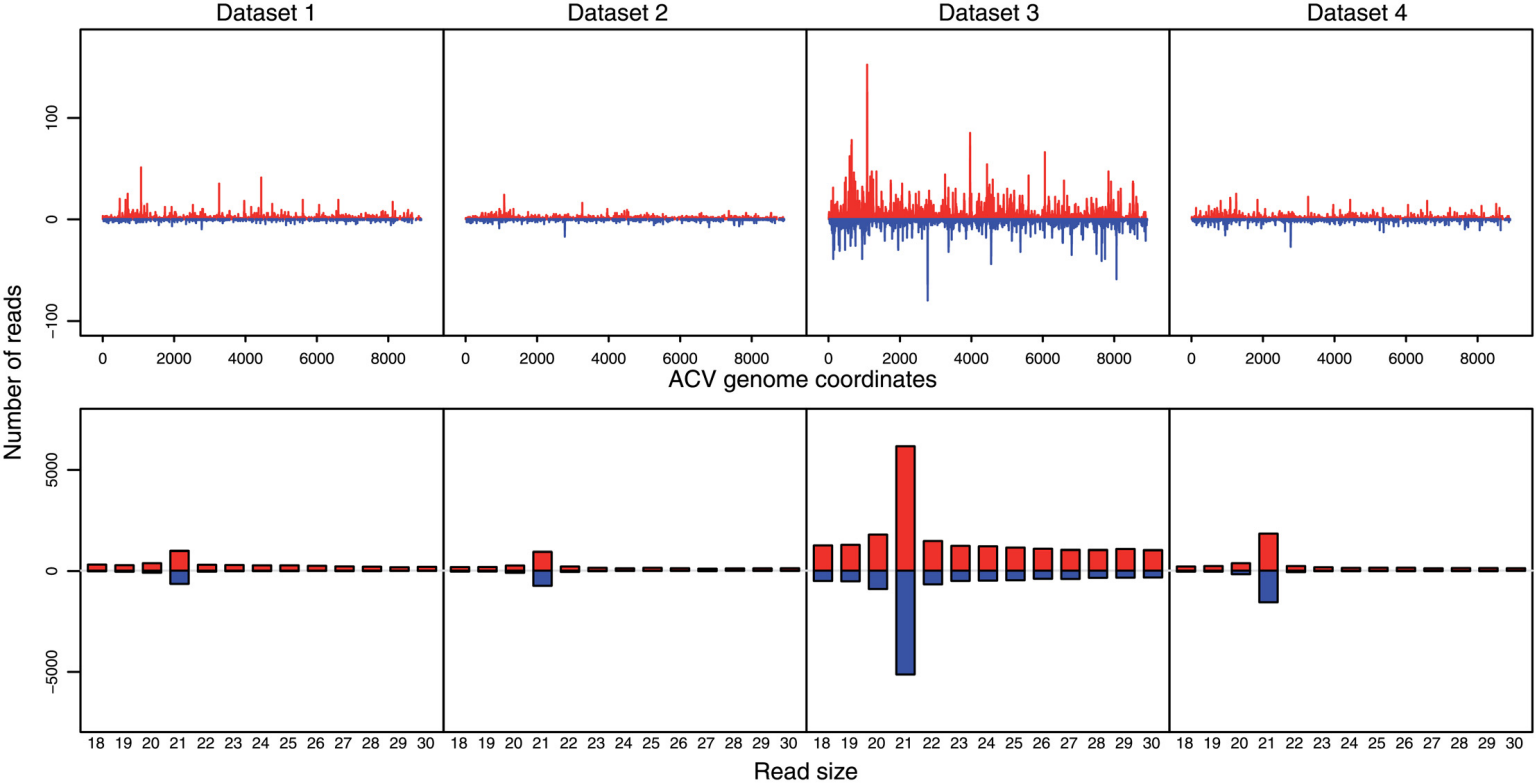
Readmaps and size distributions of small RNAs that align on the *Anopheles* C virus reconstructed genome. The four small RNA datasets were independently aligned to the sequence of the *Anopheles* C virus (AnCV) genome (accession number KU169878) with bowtie allowing no mismatches. From the bowtie output, lattice maps and read size histograms were generated representing the positions and size of the small RNAs that align to the viral genome.

Realignment of the small RNA reads on the reconstructed genome revealed, as for AnCPV, a dicing profile of virus replication intermediates, yielding virus-derived siRNAs with a read length peaking at 21 nt in all four datasets (Fig 4). These results are evidence for a previously unknown, actively replicating virus.

**Fig 5:**
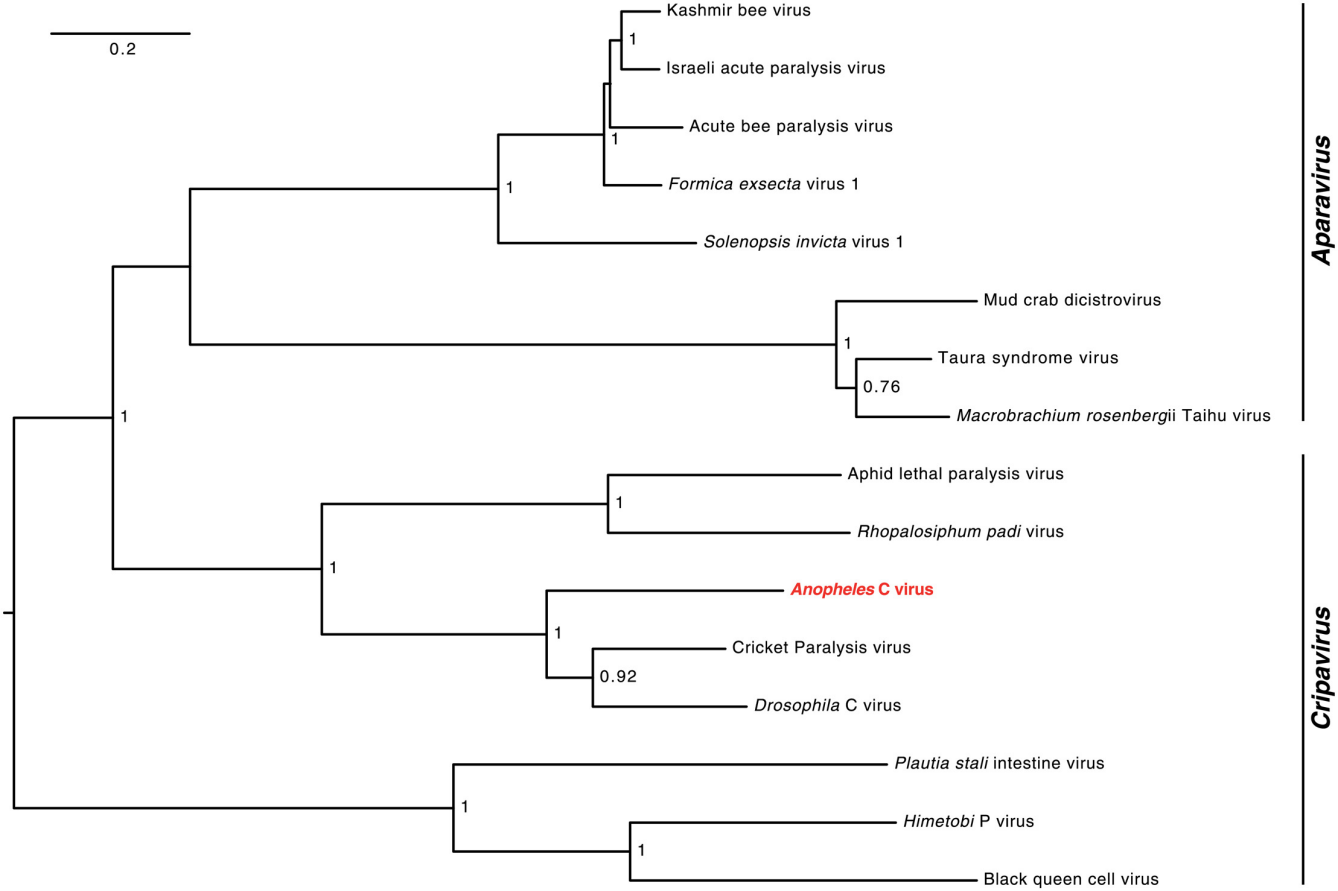
AnCV non-structural protein phylogeny. Neighbor-joining tree of non-structural polyprotein sequences of Dicistroviruses. NJ bootstrap values (above 0.75) are indicated above branches. The two genera of the *Dicistroviridae* family are indicated on the tree. Pairwise genetic distances are indicated in S6 File.

Phylogenetic tree reconstruction of non-structural proteins from *Dicistroviridae* shows that the discovered virus is related to known Dicistoviruses and groups with the *Cripavirus* genus (Fig 5). Similar to the detection of AnCPV, RT-PCR primers, designed on the reconstructed AnCV sequence, were also successfully used to generate amplicons from the total RNA used for small RNA library preparation. Sanger sequencing of the amplified region showed no variation from the bioinformatically reconstructed genomic sequence (S1 Zipped Archive). Because a paralysis phenotype was not observed in the source mosquito colony, we inferred that the virus is most likely a C-type Dicistrovirus rather than a paralysis virus and named it *Anopheles* C virus (AnCV).

**Fig 6:**
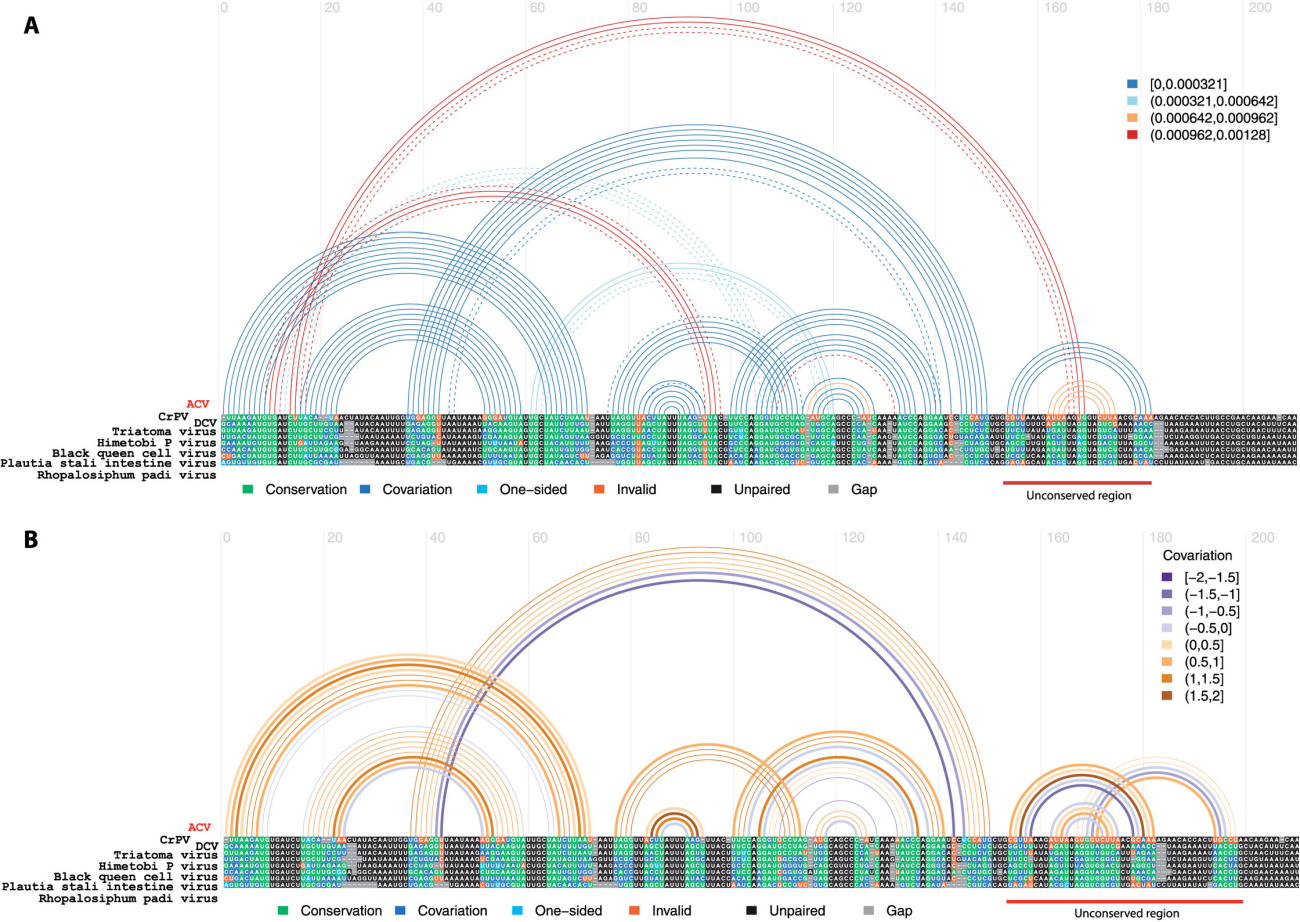
AnCV IRES show conservation of structure with the Cripavirus IRES family (RF0458). Multiple sequence alignment of AnCV potential IRES sequence (nucleotides 5800 to 6001) with the seven seed IRES of the RF0458 family. Panel (A) represents the most probable conserved helix and secondary structures of the IRES using the known RF0458 structure as a scaffold, and panel (B) depicts the covariance between the nucleotides in the known RF0458 structure, showing covariation of the nucleotides to conserve IRES structural integrity.

We investigated whether, in addition to the two ORFs at similar positions found in other Dicistroviruses, AnCV also possessed an Internal Ribosome Entry Site (IRES) of the same class and family as observed in other Dicistroviruses in order to translate the second ORF. Searching the RNA family database (Rfam) [37], we identified a 200nt region (position 5795 to 5994) of AnCV as a potential IRES, with a *p*-value of 9.4e-33 for the RF0458 CrPV IRES family (S1 File). Multiple alignment of the IRES sequences of the RF0458 family and AnCV (S2 File) showed RNA secondary structure conservation (Fig 6A) and covariance of the nucleotides within the structured pairs (Fig 6B). These results indicate that this region of the AnCV genome, situated between the two ORFs, is structurally conserved and has a high probability of being a true functional IRES, validating the result of the Rfam search.

### Cypovirus and Dicistrovirus biological evidence

**Fig 7:**
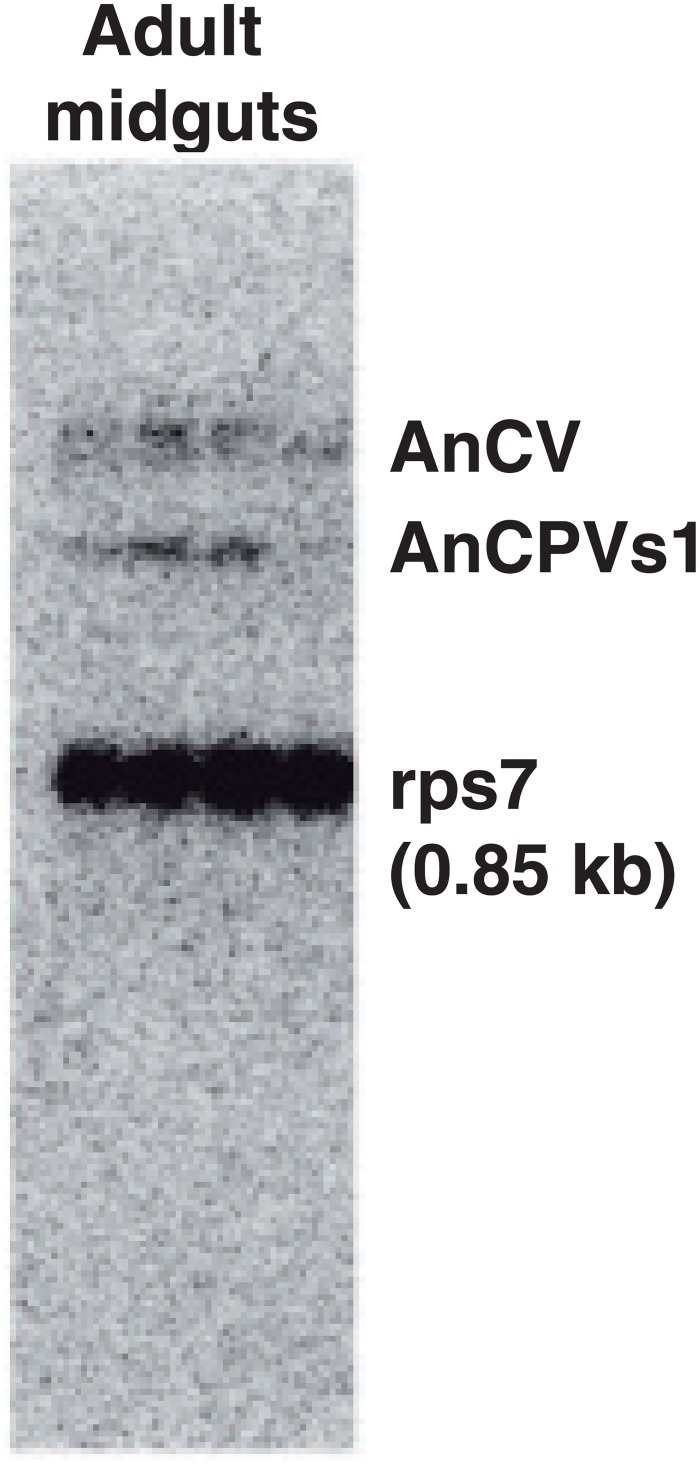
Northern blot of total RNA purified from pooled midguts of *An*. *coluzzii* adult mosquitoes shows the presence of the positive strand of AnCV genome and AnCPV segment 1.

Cloned virus fragments were used to synthesize RNA probes for hybridization to a Northern blot of the total RNA used for small RNA sequencing (Fig 7). The probes with sequence homology to the AnCV genome and the segment 1 (RdRp) of AnCPV revealed the presence of intact genomic viral RNA in the total RNA from mosquito pools for both viruses. This result also rules out RNA degradation as a source of the small RNAs detected by deep sequencing, and strengthens the interpretation of the dicing patterns (Figs 1 and 4) as a product of RNA from actively replicating virus.

In order to assess the infection prevalence and pattern in the *An*. *coluzzii* colony, we examined individual mosquitoes and individual mosquito organs by RT-PCR using virus-specific primers (Table 1). The results indicated that adult mosquito midguts, carcasses and salivary glands were susceptible to infection by both AnCPV and AnCV. Dissected tissues were washed extensively in PBS to minimize contamination, and negative controls were included.

**Table 1:** Viral prevalence in *An*. *coluzzii* Ngousso mosquitoes. Total RNA from midgut and carcass of individual mosquitoes was tested by RT-PCR for infection with AnCV and AnCPV. Salivary gland RNA was extracted from individual mosquitoes different from those used for the carcass and midgut dissection. Percent infected represents the total number of mosquitoes with infection in either carcass or midgut. ND, not done. Data available in S2 File.

	Batch 1 (2–3 days old) n = 48		Batch 2 (10 days old—1 blood meal) n = 24		Batch 3 (2–3 days old) n = 16	
	AnCV	AnCPV	AnCV	AnCPV	AnCV	AnCPV
**Infected**	81.25%	33.33%	17%	42%	ND	ND
**Midgut**	58.33%	29.17%	13%	29%	ND	ND
**Carcass**	70.83%	18.75%	13%	17%	ND	ND
**Salivary glands**	ND	ND	ND	ND	42.86%	33.33%

Mosquitoes displayed variable intensity of the RT-PCR amplification products on agarose gels. Although not a quantitative assay, this observation suggests that individual mosquitoes harbored different levels of infection. Mosquitos were infected by one, both or neither of the two viruses (S1 Table). Some mosquitoes were PCR-positive for AnCPV in the carcass but negative for the midgut, suggesting that infection in those mosquitoes might have been resolved in the midgut or that transmission might have been vertical. The reason for differences in the AnCV prevalence between batch 1 and 2 could be explained by multiple factors such as age of mosquitoes since emergence, or blood feeding status. Detailed studies will be needed to clarify the tropism and temporal dynamics of infection in mosquitoes.

### Mosquito host range and transmission

Other colonies of *An*. *coluzzii* (Fd03, Fd05, Fd09) and *An*. *gambiae* (Mbita) tested by RT-PCR were consistently infected with both viruses, but the significance of the observation is unclear because they were all maintained in the same insectaries as the Ngousso *An*. *coluzzii* colony at Institut Pasteur Paris. Samples tested from a colony of *An*. *dirus* (Pursat) maintained at Institut Pasteur Cambodia were also positive for both viruses, but the history of other insectaries where the colony was previously maintained is unknown (although it has not been kept in the Paris facility).

Interestingly, we did not detect either virus in a colony of *An*. *stephensi* (SDA500) maintained in the same Institut Pasteur Paris insectary as the *An*. *coluzzii* Ngousso colony, nor in two *Aedes aegypti* isofemale lines. Since oral transmission is the natural route of DCV infection [38] as well as of the two known mosquito Cypoviruses [30, 31], we tested whether the novel viruses are transmissible orally to these uninfected mosquito species. We reared eggs from the virus-negative *An*. *stephensi* and the two *Ae*. *aegypti* lines and divided first instar larvae of each into two trays. One tray of larvae was supplemented with homogenized *An*. *coluzzii* Ngousso fourth instar larvae, carrying AnCV and AnCPV viruses, while the other tray of larvae was fed normally and maintained in the same insectary as a control. *An*. *stephensi* and *Ae*. *aegypti* fourth instar larvae and adult females were then collected and assayed for AnCV and AnCPV as pools or individually. Larvae were washed in multiple changes of clean water and starved over several hours before processing in order to minimize the possibility of external contamination or midgut content contamination. This *per os* exposure did not infect the *Ae*. *aegypti* mosquitoes, but successfully infected *An*. *stephensi* mosquitoes with both AnCV and AnCPV at a prevalence of 59% and 100% respectively, while mosquitoes issued from the control trays remained negative (Table 2).

**Table 2:** Prevalence of infection with AnCV and AnCPV *in An*. *stephensi* adult F0 mosquitoes exposed to viruses in the larval stage. Individual adult mosquitoes were collected after feeding as larvae with extract of infected *An*. *coluzzii*, and infection with both viruses was tested by RT-PCR. Values represent the proportion of mosquitoes with infection in indicated tissues.

	AnCV	AnCPV
**Total infected (n = 90)**	58.89%	100.00%
**Midgut (n = 90)**	26.67%	100.00%
**Carcass (n = 90)**	46.67%	73.33%

To assess whether the viruses can be transmitted vertically, *An*. *stephensi* adult females developed from larvae experimentally infected as above (the F0 generation) were allowed to blood feed and lay eggs (F1 generation). Both F1 larvae as well as resulting adult females displayed AnCPV and AnCV infections (Table 3). These results suggest that both of the viruses can be transmitted vertically to the progeny by oviposition. *An*. *stephensi* mosquitoes maintained until the F4 generation after virus exposure displayed persistent infection with AnCPV, but AnCV prevalence decreased each generation and was absent in F4 adults.

**Table 3:** Prevalence of infection with AnCV and AnCPV in *An*. *stephensi* F1 generation mosquitoes. Individual larvae or adult mosquitoes were collected the generation after exposure to viruses, and infection with both viruses was tested by RT-PCR.

	AnCV	AnCPV
**Larvae infected (n = 22)**	36.36%	63.63%
**Adults infected (n = 24)**	8.33%	66.67%

### Virus presence in wild Anopheles mosquitoes

**Fig 8:**
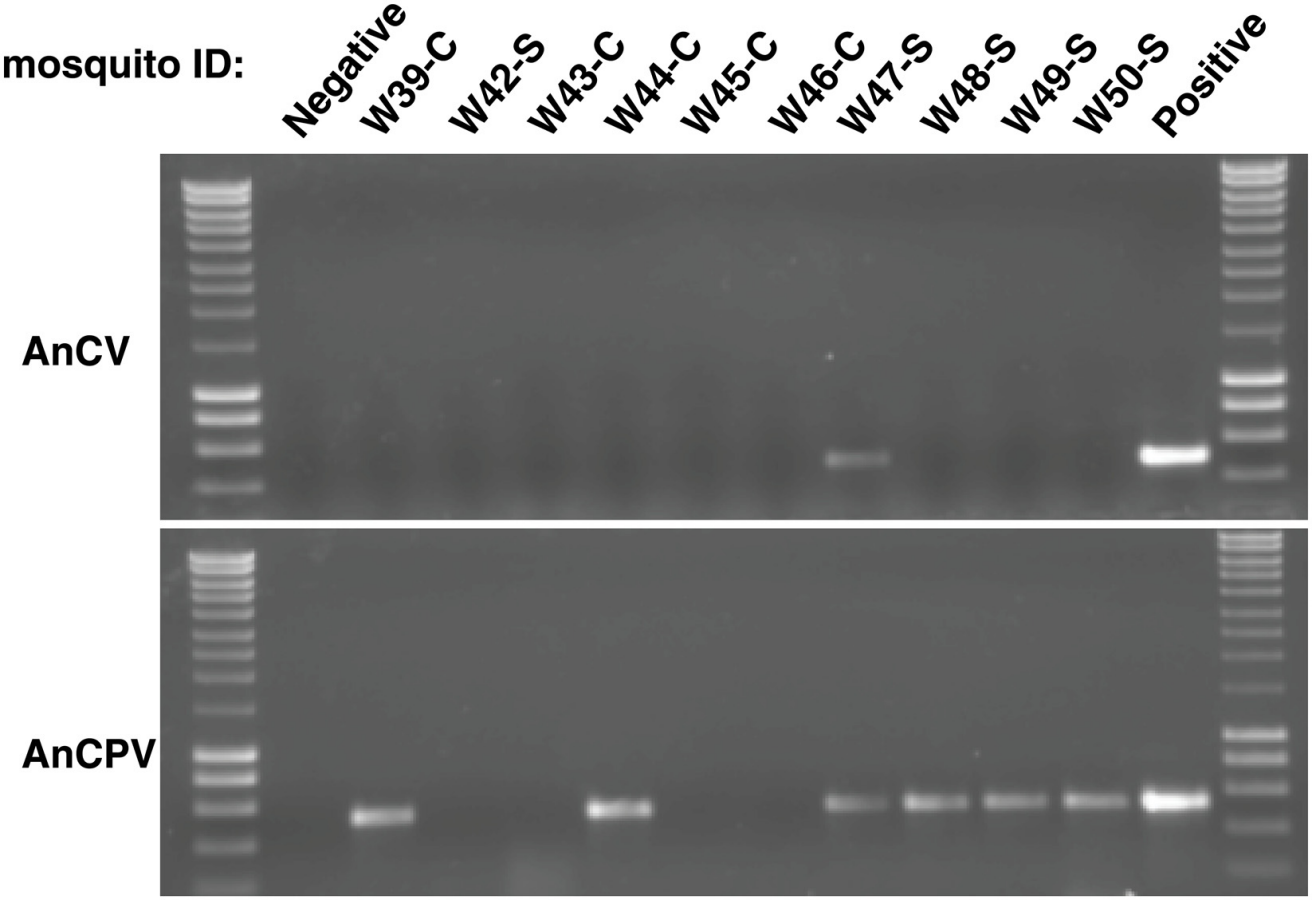
Example of RT-PCR amplicons of AnCV and AnCPV non-structural regions separated by agarose gel for wild mosquitoes and positive and negative controls. Mosquito ID suffix -S or -C stands for the country of collection, Senegal or Cambodia respectively.

We captured wild *Anopheles spp*. mosquitoes in villages in Senegal and Cambodia, and tested individual mosquito RNAs for the viruses using specific RT-PCR. Bands of the correct predicted sizes were detected in some of the samples: 494nt for AnCV and 524nt for AnCPV (Fig 8). Samples were processed along with negative controls in order to control for cross-contamination. Individual wild mosquitoes were positive for one, both, or neither virus.

One wild *Anopheles spp*. from Senegal was positive for AnCV, and six wild *Anopheles spp*. from Senegal and Cambodia were positive for AnCPV. The resulting amplicons were sequenced in both orientations. In addition, AnCV amplicons were detected in two *An*. *dirus* mosquitoes from the Cambodia insectary colony, and were also sequenced. Alignment of these sequences with the reconstructed consensus sequences showed high sequence homology with a maximum of three SNPs for AnCPV and one SNP for AnCV in the analyzed region (S3 and S4 Figs). The results indicate that AnCV is present in the natural virome of wild *Anopheles* mosquitoes from at least Africa, and AnCPV is present in the natural virome of wild *Anopheles* mosquitoes from Africa and Asia.

## Discussion

By deep sequencing of small RNAs we have identified two novel insect viruses, a Cypovirus and a Dicistrovirus, in a laboratory colony of *An*. *coluzzii* mosquitoes. Reconstructing viral genomes from viral-derived small RNAs enriches for detection of actively replicating viruses. We present biological evidence for the presence of these viruses as a persistent infection in colonies of three species of *Anopheles* (*An*. *coluzzii*, *An*. *gambiae*, *An*. *dirus*) and their exclusion from a colony of one other species (*An*. *stephensi*). Because of the possibility of horizontal transmission in the insectary, data from colonies are not informative for infection in nature, but it does at least indicate the species range of permissive hosts. Importantly, the viruses were present in wild *Anopheles* mosquitoes captured in Senegal and Cambodia, thus establishing them as components of the natural virome.

We identified *Anopheles* Cypovirus (AnCPV), which contains a genomic segment coding for the polyhedrin protein characteristic of this viral family and a genomic segment coding for an RNA-dependent RNA polymerase that shows conservation with the genomes of known Cypoviruses. This is, to our knowledge, the first reported member of the *Cypoviridae* family infecting *Anopheles* mosquitoes, complementing previous studies identifying Cypoviruses in other mosquito genera [30, 31]. We also identified an *Anopheles* C virus (AnCV) that displays genetic architecture consistent with other members of the family *Dicistroviridae*, with two open reading frames, 5’ non-structural polyprotein and 3’ structural polyprotein, as well as conserved RNA secondary structure and sequence of the central IRES. This virus was further confirmed to belong to the *Dicistroviridae* family based on the amino-acid sequence conservation of the AnCV RNA-dependent RNA polymerase with its orthologs of the *Drosophila* C virus (DCV) and Cricket paralysis virus (CrPV).

The novel Dicistrovirus AnCV can infect some *Anopheles* but not *Aedes* mosquitoes, and appears not to induce high mortality nor detectable paralysis phenotypes in mosquitoes infected *per os*. These data suggest that infection phenotype and range of this virus resemble that of DCV and not CrPV. We cannot exclude that in our model, the virus and the laboratory colony could have co-evolved to minimize pathogenic symptoms. The virus-negative *An*. *stephensi* colony, when infected with AnCV *per os*, also did not display detectable pathogenic symptoms. This scenario could be tested with isolated viruses by infection experiments using different concentrations of virus to study symptoms and pathogenicity. Isolated and culturable viruses would also allow studies of virion morphology, mechanisms of entry, host range, obtaining complete genomic sequences, and generating infectious clones. The main mosquito organs were found to be positive for both viruses. Development of a quantitative real-time PCR assay would be useful in order to measure genome copies in different organs.

DCV, first isolated in 1972 [39], and CrPV are models for the study of insect antiviral immunity. These viruses as well as other members of the family are ecologically and agriculturally relevant [40]. The discovery of a virus from this family in laboratory and wild anopheline mosquitoes is an important first step to understanding the natural viral population in these major human malaria vectors, and potentially establishing a viral infection model in those mosquitoes.

It will be interesting to determine whether AnCV has similar phenotypes for *Anopheles* mosquitoes as the model insect virus DCV has for *Drosophila*. For example, DCV leads to a strong immune response and to fly death within several days when intra-thoracically injected [41], but produces only mild effects after infection by the oral route, with transcriptional induction of only 11 genes [42]. Although the small transcriptional response may suggest a co-evolved state, DCV has also been associated with population crashes and shorter longevity of some laboratory colonies, even in persistently infected fly stocks [40].

The first infectious clone of a Dicistrovirus, CrPV [43], was recently generated, which suggests that an AnCV infectious clone as a tool is within reach. This would open an array of possibilities to study RNA virus antiviral mechanisms in the malaria vector mosquito under low-biosecurity conditions. The discovery of the first RNA viruses in the *An*. *gambiae* species complex is an initial step that will facilitate work on mosquito-virus interactions and anti-viral immunity, including the influence of the microbiota as well as potential reciprocal interactions with other pathogens such as malaria parasites.

## Materials and Methods

### Mosquito colonies

Deep sequencing of small RNAs used mosquitoes from the *An*. *coluzzii* Ngousso colony previously established from Yaoundé, Cameroon [44], and formerly described as *An*. *gambiae* M molecular form. Other colonies used and raised in the Institut Pasteur Paris insectaries were *An*. *gambiae* Mbita from Kenya (obtained from the ICIPE Field Station, Mbita Point, Kenya), Fd03 initiated from Mali, Fd05 and Fd09 from Burkina Faso, and *An*. *stephensi* colony SDA500. In addition, two *Aedes aegypti* isofemale lines from Thailand were used [45]. All mosquitoes were reared under standard conditions at 26°C and 80% relative humidity, with a 12 h light/dark cycle as previously described [44]. Finally, mosquito samples from the *An*. *dirus* Pursat colony reared in the insectaries of Institut Pasteur Cambodia, and never grown at Institut Pasteur Paris, were also used.

### Wild mosquitoes

Wild *Anopheles* mosquitoes were used for testing of virus presence in nature. Mosquitoes were captured in Ratanakiri and Kampong Chnang, Cambodia and Kedougou, Senegal were visually identified as *Anopheles spp*., and immediately transferred into RNAlater at 4°C, and then stored at -80°C. Permission to collect mosquitoes was obtained by Institut Pasteur Cambodia from authorities of Ratanakiri and Kampong Chnang, and by Institut Pasteur Dakar from authorities of Kedougou. RNA was extracted from individual mosquitoes using the Nucleospin miRNA kit (Macherey-Nagel) following the supplied protocol. RT-PCR assays for AnCV and AnCPV amplicons were performed as described below (using 45 PCR cycles), with blank reactions and negative control RNA samples. Amplicons from positive samples were Sanger sequenced and aligned with virus sequences assembled from deep sequencing of *An*. *coluzzii* Ngousso mosquito RNA. SNPs were verified using the trace files.

### RNA preparation and deep sequencing

Pools of 30 *An*. *coluzzii* Ngousso colony mosquito midguts were homogenized in TRIzol (Ambion) and total RNA was extracted following the supplied protocol. Small-RNA libraries were prepared from the small RNA fraction using TruSeq Small RNA Library reagents (Illumina). Libraries were sequenced on an Illumina Hiseq 2500 in a multiplexed 51 +7 bases single read using a TruSeq SR cluster kit v3 cBot HS, and a TruSeq SBS kit v3 HS 50 cycles (Illumina). Primary analysis of the sequences was performed with Casava software (v1.8 Illumina). Library preparation and sequencing was performed by ARK Genomics at the Roslin Institute (University of Edinburgh). Data are available in the EBI European Nucleotide Archive (http://www.ebi.ac.uk/ena/) under study accession number ERP012577 and sample accession numbers: ERX1180340, ERX1180341, ERX1180342 and ERX1180343.

Long paired-end RNA libraries were prepared and sequenced by the Institut Pasteur Transcriptomics and Epigenomics core facility using standard long paired-end rapid run protocol on an Illumina Hiseq 2500. Primary analysis of the sequences was performed with Casava software (v1.8 Illumina). Long paired-end sequencing data are available in the EBI European Nucleotide Archive (http://www.ebi.ac.uk/ena/) under accession numbers: ERX1220645 and ERX1220646.

### Bioinformatic virus discovery

Bioinformatic analysis was performed with the Galaxy [29, 46, 47] Mississippi server instance hosted by the ARTbio facility using the Metavisitor workflows (v1.0) [28]. Briefly, small RNA sequencing reads were clipped of the adapter sequence, converted to fasta format and concatenated. Then reads were converted to a weighted fasta format to reduce computing load. Reads were then depleted by successive bowtie alignments on the genomes of the known organisms present in the dataset. Unaligned unique reads were then used for the subsequent contig assembly. Velvet and Oases were used to reconstruct longer contigs with k-mers ranging from 15 to 29 [48, 49]. The NCBI BLAST tool suite was used to search a BLAST database created from the nucleotide sequences deposited in the NCBI repository with the reconstructed virus contigs, for related homologies. To reconstruct a more complete AnCV genome, an additional dataset from long paired-end sequencing was used (ERS977505). Similarly, Metavisitor was used but with longer k-mers (ranging from 25 to 69). These analyses are accessible in the Mississippi Galaxy server using the link to a Galaxy page https://mississippi.snv.jussieu.fr/u/drosofff/p/anopheles-viruses.

### IRES structure analysis

Using the structure of the IRES RF0458 family as a scaffold and the multiple alignment between the seeds of this IRES family and the potential IRES sequence of AnCV (S2 File, we used webserver based versions of TRANSAT to plot the most probable conserved helix and secondary structures of the IRES [50](Fig 6A) (http://www.e-rna.org/transat), and R-chie (http://www.e-rna.org/r-chie) in “single covariance” mode to plot the covariance between the nucleotides in the structure [51](Fig 6B). Both programs were used with the defaults parameters and with the structure and multiple alignments given in S2 File as inputs.

### RT-PCR assays for detection of virus presence

RNA from individual mosquitoes, individual organs, saliva or pools of either was extracted using either TRIzol (Ambion), NucleoSpin RNA II or NucleoSpin 96 RNA kits (Macherey-Nagel) according to the supplied protocol. RT-PCR was performed using Superscript One-Step RT-PCR kit (Invitrogen), primer (S2 Table) annealing was performed at 56°C. For the sequencing of PCR amplicons from wild or colony mosquitoes, amplicons were purified using the QIAquick PCR Purification kit following manufacturer instructions. Sanger sequencing was performed using T7 primers by SEGENIC Eurofins, Cochin Institute, Paris.

### Northern blotting

RNA migration and Northern blotting used standard procedures. Northern blot sense and antisense probes were synthesized by SP6 transcription of the PCR fragments incorporated in the PGEM-t easy vector. Multiple vector clones were verified by classical Sanger sequencing and no variation from the *in silico* reconstructed contigs was observed. RNA probe hybridization to the Northern blot was performed at 68°C.

### Phylogenetic analysis

Basic multiple alignment of the polyhedrin segment sequences was performed using Clustal O (v1.2.1) and sequence accession numbers DQ212785.1 and AY876384.1. Basic amino acid comparison for the polyhedrin protein of AnCPV (segment 10, KU169880) was performed against ABA61432.1 and AAW80667.1 using Serial Cloner (v2.6.1, http://serialbasics.free.fr).

MUSCLE multiple alignment and evolutionary analyses were conducted in MEGA (Tamura et al., 2011) with 1000 bootstrap replicates. Because non-conserved regions could possibly influence the phylogenetic tree reconstructions, gaps were removed using the 'Complete Deletion' option in MEGA. Phylogenetic reconstructions using neighbor joining (NJ), maximum parsimony (MP) or maximum likelihood (ML) showed the same topology for each dataset. Trees constructed with partial deletion are also included (S2 Fig). EMBL-EBI Transeq and Sixpack online tools (http://www.ebi.ac.uk/Tools/st/) were used to translate nucleic acid sequences to the corresponding peptide sequences.

The multiple alignments for Fig 2 and S1 Fig are based on the amino acid sequences given in S3 and S4 Files.

Accession numbers for the RdRp alignment: YP_009111328.1 (*Dendrolimus punctatus* cypovirus 22), NP_149147.1 (*Lymantria dispar* cypovirus 1), ABB51571.1 (*Heliothis armigera* cypovirus 14), AF291683_3 (*Trichoplusia ni* cytoplasmic polyhedrosis virus 15), DQ192251.1_1 (*Operophtera brumata* cypovirus 19), AJC97790.1 (*Thaumetopoea pityocampa* cypovirus 5), AHJ14791.1 (*Inachis io* cypovirus 2), ACA53380.1 (*Choristoneura occidentalis* cypovirus 16), ADH10220.1 (*Antheraea mylitta* cypovirus 4).

Accession numbers for the polyhedrin alignment: AAA50751.1 (*Heliothis armigera* cypovirus 8), AJC97797.1 (*Thaumetopoea pityocampa* cypovirus 5), NP_066381.1 (*Trichoplusia ni* cypovirus 15), ABB17224.1 (*Operophtera brumata* cypovirus 19), AAY96324.1 (*Heliothis assulta* cypovirus 14), ACX54964.1 (*Bombyx mori* cypovirus 1), ABB17220.1 (*Operophtera brumata* cypovirus 18), AAW80667.1 (*Uranotaenia sapphirina* cypovirus 17), YP_009002596.1 (*Inachis io* cypovirus 2), ABW87644.1 (*Choristoneura occidentalis* cypovirus 16), ABH85367.1 (*Simulium ubiquitum* cypovirus 20), AAP44510.1 (*Antheraea proylei* cypovirus 4), AIY60608.1 (*Dendrolimus punctatus* cypovirus 22).

The amino acid sequences used for the multiple alignments for Fig 5 and S2 Fig are given in S4 Fig. Accession numbers for the amino acid sequences used: ALS55295 (*Anopheles* C virus), NP_647481.1 (Cricket Paralysis virus), NP_044945.1 (*Drosophila* C virus), AF486073_1 (Acute bee paralysis virus), YP_008888535.1 (*Formica exsecta* virus 1), YP_164440.1 (*Solenopsis invicta* virus 1), NP_851403.1 (Kashmir bee virus), ABY57949.1 (Israeli acute paralysis virus), NP_620555.1 (*Plautia stali* intestine virus), AGF84786.1 (Aphid lethal paralysis virus), AAT81157.2 (Taura syndrome virus), NP_046155.1 (*Rhopalosiphum padi* virus), YP_006666503.1 (*Macrobrachium rosenbergii* Taihu virus), YP_004063985.1 (Mud crab dicistrovirus), AGW80519.1 (*Himetobi* P virus), NP_620564.1 (Black queen cell virus).

## Supporting Information

S1 Fig AnCPV RNA dependent RNA polymerase and polyhedrin phylogeny. (A) Maximum likelihood tree of RNA-dependent RNA polymerase (RdRp) sequences of Cypoviruses, (B) Maximum likelihood tree of Polyhedrin sequences of Cypoviruses. Multiple protein alignment was done using the partial deletion option in MEGA. ML bootstrap values (above 0.75) are indicated above branches.
(PDF)

S2 Fig AnCV non-structural protein phylogeny. Neighbor-joining tree of non-structural polyprotein sequences of Dicistrovirus. Multiple protein alignment was done using the partial deletion option in MEGA. NJ bootstrap values above 0.75 are indicated above branches. The two genera of the *Dicistroviridae* family are indicated on the tree.
(PDF)

S3 Fig Multiple alignments of AnCPV sequences from wild caught mosquitoes and controls. F and R suffix represent respectively forward and reverse sequencing orientations. Sanger sequencing raw files are in S3 Zipped Archive.
(PDF)

S4 Fig Multiple alignments of AnCV sequences from wild caught mosquitoes and controls. F and R suffix represent respectively forward and reverse sequencing orientations. Sanger sequencing raw files are in S3 Zipped Archive.
(PDF)

S1 File Rfam search details xml file. (XML)

S2 File Structure of the RF00458 IRES family followed by the ClustalW multiple sequence alignment of AnCV potential IRES sequence (nucleotides 5800 to 6001) with the seven seed IRES of the RF0458 family. These are the inputs for the R-Chie and TRANSAT online tools.
(TXT)

S3 File Muscle alignment file for the RdRp of Cypoviruses. (TXT)

S4 File Muscle alignment file for the polyhedrin of Cypoviruses. (TXT)

S5 File Muscle alignment file for the RdRp of Dicistroviruses. (TXT)

S6 File Pairwise genetic distances for the different phylogenetic trees. (XLSX)

S1 Table Detailed RT-PCR results of mosquitoes. Ones and zeros highlight RT-PCR positive and negative results, respectively. Yellow color represents AnCV and AnCPV negative mosquitoes while blue and green colors highlight mosquitoes positive only in the midgut or carcass for either virus.
(XLSX)

S2 Table Primers used for one step RT-PCR. Final suffix indicates forward, F, or reverse, R, sense of primers.
(DOCX)

S1 Zipped Archive Compressed file containing raw Sanger sequencing results for the RT-PCR viral fragments. (ZIP)

## References

[1] Li CX, Shi M, Tian JH, Lin XD, Kang YJ, Chen LJ, et al Unprecedented genomic diversity of RNA viruses in arthropods reveals the ancestry of negative-sense RNA viruses. Elife. 2015;4 10.7554/eLife.05378 25633976 PMC4384744

[2] Huhtamo E, Cook S, Moureau G, Uzcategui NY, Sironen T, Kuivanen S, et al Novel flaviviruses from mosquitoes: mosquito-specific evolutionary lineages within the phylogenetic group of mosquito-borne flaviviruses. Virology. 2014;464–465:320–9. 10.1016/j.virol.2014.07.015 25108382 PMC4170750

[3] Zuo S, Zhao Q, Guo X, Zhou H, Cao W, Zhang J. Detection of Quang Binh virus from mosquitoes in China. Virus Res. 2014;180:31–8. 10.1016/j.virusres.2013.12.005 . 24342141

[4] Nasar F, Palacios G, Gorchakov RV, Guzman H, Da Rosa AP, Savji N, et al Eilat virus, a unique alphavirus with host range restricted to insects by RNA replication. Proc Natl Acad Sci U S A. 2012;109(36):14622–7. Epub 2012/08/22. 10.1073/pnas.1204787109 22908261 PMC3437828

[5] Vasilakis N, Forrester NL, Palacios G, Nasar F, Savji N, Rossi SL, et al Negevirus: a proposed new taxon of insect-specific viruses with wide geographic distribution. J Virol. 2013;87(5):2475–88. 10.1128/JVI.00776-12 23255793 PMC3571365

[6] Cook S, Chung BY, Bass D, Moureau G, Tang S, McAlister E, et al Novel virus discovery and genome reconstruction from field RNA samples reveals highly divergent viruses in dipteran hosts. PloS one. 2013;8(11):e80720 10.1371/journal.pone.0080720 24260463 PMC3832450

[7] Webster CL, Waldron FM, Robertson S, Crowson D, Ferrari G, Quintana JF, et al The Discovery, Distribution, and Evolution of Viruses Associated with Drosophila melanogaster. PLoS Biol. 2015;13(7):e1002210 Epub 2015/07/15. 10.1371/journal.pbio.1002210 26172158 PMC4501690

[8] Myles KM, Morazzani EM, Adelman ZN. Origins of alphavirus-derived small RNAs in mosquitoes. RNA Biol. 2009;6(4):387–91. Epub 2009/06/19. 8946 [pii]. 19535909 10.4161/rna.6.4.8946 PMC2811051

[9] Myles KM, Wiley MR, Morazzani EM, Adelman ZN. Alphavirus-derived small RNAs modulate pathogenesis in disease vector mosquitoes. Proc Natl Acad Sci U S A. 2008;105(50):19938–43. Epub 2008/12/03. 10.1073/pnas.0803408105 19047642 PMC2604946

[10] Siu RW, Fragkoudis R, Simmonds P, Donald CL, Chase-Topping ME, Barry G, et al Antiviral RNA interference responses induced by Semliki Forest virus infection of mosquito cells: characterization, origin, and frequency-dependent functions of virus-derived small interfering RNAs. J Virol. 2011;85(6):2907–17. Epub 2010/12/31. 10.1128/JVI.02052-10 21191029 PMC3067965

[11] Hess AM, Prasad AN, Ptitsyn A, Ebel GD, Olson KE, Barbacioru C, et al Small RNA profiling of Dengue virus-mosquito interactions implicates the PIWI RNA pathway in anti-viral defense. BMC Microbiol. 2011;11:45 Epub 2011/03/02. 10.1186/1471-2180-11-45 21356105 PMC3060848

[12] Morazzani EM, Wiley MR, Murreddu MG, Adelman ZN, Myles KM. Production of virus-derived ping-pong-dependent piRNA-like small RNAs in the mosquito soma. PLoS Pathog. 2012;8(1):e1002470 Epub 2012/01/14. 10.1371/journal.ppat.1002470 22241995 PMC3252369

[13] Scott JC, Brackney DE, Campbell CL, Bondu-Hawkins V, Hjelle B, Ebel GD, et al Comparison of dengue virus type 2-specific small RNAs from RNA interference-competent and -incompetent mosquito cells. PLoS Negl Trop Dis. 2010;4(10):e848 Epub 2010/11/05. 10.1371/journal.pntd.0000848 21049014 PMC2964303

[14] Carissimo G, Pondeville E, McFarlane M, Dietrich I, Mitri C, Bischoff E, et al Antiviral immunity of Anopheles gambiae is highly compartmentalized, with distinct roles for RNA interference and gut microbiota. Proc Natl Acad Sci U S A. 2015;112(2):E176–85. 10.1073/pnas.1412984112 25548172 PMC4299212

[15] Kreuze JF, Perez A, Untiveros M, Quispe D, Fuentes S, Barker I, et al Complete viral genome sequence and discovery of novel viruses by deep sequencing of small RNAs: a generic method for diagnosis, discovery and sequencing of viruses. Virology. 2009;388(1):1–7. 10.1016/j.virol.2009.03.024 . 19394993

[16] Wu Q, Luo Y, Lu R, Lau N, Lai EC, Li WX, et al Virus discovery by deep sequencing and assembly of virus-derived small silencing RNAs. Proc Natl Acad Sci U S A. 2010;107(4):1606–11. 10.1073/pnas.0911353107 20080648 PMC2824396

[17] Bell-Sakyi L, Kohl A, Bente DA, Fazakerley JK. Tick cell lines for study of Crimean-Congo hemorrhagic fever virus and other arboviruses. Vector Borne Zoonotic Dis. 2012;12(9):769–81. 10.1089/vbz.2011.0766 21955214 PMC3438810

[18] Ciota AT, Payne AF, Ngo KA, Kramer LD. Consequences of in vitro host shift for St. Louis encephalitis virus. J Gen Virol. 2014;95(Pt 6):1281–8. 10.1099/vir.0.063545-0 24643879 PMC4027038

[19] Sudeep AB, Parashar D, Jadi RS, Basu A, Mokashi C, Arankalle VA, et al Establishment and characterization of a new Aedes aegypti (L.) (Diptera: Culicidae) cell line with special emphasis on virus susceptibility. In Vitro Cell Dev Biol Anim. 2009;45(9):491–5. 10.1007/s11626-009-9218-1 . 19533252

[20] Posey DL, O'Rourke T, Roehrig JT, Lanciotti RS, Weinberg M, Maloney S. O'Nyong-nyong fever in West Africa. Am J Trop Med Hyg. 2005;73(1):32 . 16014827

[21] Powers AM, Brault AC, Shirako Y, Strauss EG, Kang W, Strauss JH, et al Evolutionary relationships and systematics of the alphaviruses. J Virol. 2001;75(21):10118–31. 10.1128/JVI.75.21.10118-10131.2001 11581380 PMC114586

[22] Coffey LL, Failloux AB, Weaver SC. Chikungunya virus-vector interactions. Viruses. 2014;6(11):4628–63. 10.3390/v6114628 25421891 PMC4246241

[23] Waldock J, Olson KE, Christophides GK. Anopheles gambiae antiviral immune response to systemic O'nyong-nyong infection. PLoS Negl Trop Dis. 2012;6(3):e1565 Epub 2012/03/20. 10.1371/journal.pntd.0001565 22428080 PMC3302841

[24] Rider MA, Zou J, Vanlandingham D, Nuckols JT, Higgs S, Zhang Q, et al Quantitative proteomic analysis of the Anopheles gambiae (Diptera: Culicidae) midgut infected with o'nyong-nyong virus. J Med Entomol. 2013;50(5):1077–88. . 24180113 10.1603/me12155

[25] Sim C, Hong YS, Vanlandingham DL, Harker BW, Christophides GK, Kafatos FC, et al Modulation of Anopheles gambiae gene expression in response to o'nyong-nyong virus infection. Insect Mol Biol. 2005;14(5):475–81. 10.1111/j.1365-2583.2005.00578.x 16164603 PMC3840949

[26] Sim C, Hong YS, Tsetsarkin KA, Vanlandingham DL, Higgs S, Collins FH. Anopheles gambiae heat shock protein cognate 70B impedes o'nyong-nyong virus replication. BMC Genomics. 2007;8:231 10.1186/1471-2164-8-231 17625007 PMC1963456

[27] Keene KM, Foy BD, Sanchez-Vargas I, Beaty BJ, Blair CD, Olson KE. RNA interference acts as a natural antiviral response to O'nyong-nyong virus (Alphavirus; Togaviridae) infection of Anopheles gambiae. Proc Natl Acad Sci U S A. 2004;101(49):17240–5. 10.1073/pnas.0406983101 15583140 PMC535383

[28] ARTbio bioinformatics analysis facility—Institut de Biologie Paris Seine. Metavisitor Suite—Mississippi Tools for Virus Detection or Discovery 2015 [19 December 2015]. Available: https://toolshed.g2.bx.psu.edu/repository?repository_id=888637eebdd37d62&changeset_revision=8a55ab5f0f81.

[29] Blankenberg D, Von Kuster G, Coraor N, Ananda G, Lazarus R, Mangan M, et al Galaxy: a web-based genome analysis tool for experimentalists. Curr Protoc Mol Biol. 2010;Chapter 19:Unit 19 0 1–21. 10.1002/0471142727.mb1910s89 20069535 PMC4264107

[30] Green TB, Shapiro A, White S, Rao S, Mertens PP, Carner G, et al Molecular and biological characterization of a Cypovirus from the mosquito Culex restuans. J Invertebr Pathol. 2006;91(1):27–34. Epub 2005/12/27. 10.1016/j.jip.2005.10.007 . 16376932

[31] Shapiro A, Green T, Rao S, White S, Carner G, Mertens PP, et al Morphological and molecular characterization of a Cypovirus (Reoviridae) from the mosquito Uranotaenia sapphirina (Diptera: Culicidae). J Virol. 2005;79(15):9430–8. Epub 2005/07/15. 10.1128/JVI.79.15.9430-9438.2005 16014906 PMC1181557

[32] Sievers F, Higgins DG. Clustal Omega, accurate alignment of very large numbers of sequences. Methods Mol Biol. 2014;1079:105–16. 10.1007/978-1-62703-646-7_6 . 24170397

[33] Wang XH, Aliyari R, Li WX, Li HW, Kim K, Carthew R, et al RNA interference directs innate immunity against viruses in adult Drosophila. Science. 2006;312(5772):452–4. 10.1126/science.1125694 16556799 PMC1509097

[34] Virus Taxonomy: 2014 Release [Internet]. 2016 [cited 2 March 2016]. Available: http://www.ictvonline.org/virusTaxonomy.asp?taxnode_id=20143546.

[35] Zhou Y, Qin T, Xiao Y, Qin F, Lei C, Sun X. Genomic and biological characterization of a new cypovirus isolated from Dendrolimus punctatus. PloS one. 2014;9(11):e113201 10.1371/journal.pone.0113201 25419713 PMC4242531

[36] Bonning BC, Miller WA. Dicistroviruses. Annu Rev Entomol. 2010;55:129–50. 10.1146/annurev-ento-112408-085457 . 19961327

[37] Nawrocki EP, Burge SW, Bateman A, Daub J, Eberhardt RY, Eddy SR, et al Rfam 12.0: updates to the RNA families database. Nucleic acids research. 2015;43(Database issue):D130–7. Epub 2014/11/14. 10.1093/nar/gku1063 25392425 PMC4383904

[38] Stevanovic AL, Johnson KN. Infectivity of Drosophila C virus following oral delivery in Drosophila larvae. J Gen Virol. 2015;96(Pt 6):1490–6. 10.1099/vir.0.000068 . 25626683

[39] Jousset FX. [Iota virus of Drosophila immigrans studied in D. melanogaster: CO 2 sensitivity symptom, description of abnromalities induced in the host]. Annales de l'Institut Pasteur. 1972;123(2):275–88. . 4632292

[40] Bonning BC. The Dicistroviridae: An emerging family of invertebrate viruses. Virol Sin. 2009;24(5):415–27. 10.1007/s12250-009-3044-1

[41] Dostert C, Jouanguy E, Irving P, Troxler L, Galiana-Arnoux D, Hetru C, et al The Jak-STAT signaling pathway is required but not sufficient for the antiviral response of drosophila. Nature immunology. 2005;6(9):946–53. 10.1038/ni1237 . 16086017

[42] Roxstrom-Lindquist K, Terenius O, Faye I. Parasite-specific immune response in adult Drosophila melanogaster: a genomic study. EMBO reports. 2004;5(2):207–12. 10.1038/sj.embor.7400073 14749722 PMC1298984

[43] Kerr CH, Wang QS, Keatings K, Khong A, Allan D, Yip CK, et al The 5' untranslated region of a novel infectious molecular clone of the dicistrovirus cricket paralysis virus modulates infection. J Virol. 2015;89(11):5919–34. 10.1128/JVI.00463-15 25810541 PMC4442438

[44] Harris C, Lambrechts L, Rousset F, Abate L, Nsango SE, Fontenille D, et al Polymorphisms in Anopheles gambiae immune genes associated with natural resistance to Plasmodium falciparum. PLoS Pathog. 2010;6(9):e1001112 Epub 2010/09/24. 10.1371/journal.ppat.1001112 20862317 PMC2940751

[45] Fansiri T, Fontaine A, Diancourt L, Caro V, Thaisomboonsuk B, Richardson JH, et al Genetic mapping of specific interactions between Aedes aegypti mosquitoes and dengue viruses. PLoS Genet. 2013;9(8):e1003621 Epub 2013/08/13. 10.1371/journal.pgen.1003621 23935524 PMC3731226

[46] Giardine B, Riemer C, Hardison RC, Burhans R, Elnitski L, Shah P, et al Galaxy: a platform for interactive large-scale genome analysis. Genome research. 2005;15(10):1451–5. 10.1101/gr.4086505 16169926 PMC1240089

[47] Goecks J, Nekrutenko A, Taylor J, Galaxy T. Galaxy: a comprehensive approach for supporting accessible, reproducible, and transparent computational research in the life sciences. Genome Biol. 2010;11(8):R86 10.1186/gb-2010-11-8-r86 20738864 PMC2945788

[48] Zerbino DR, Birney E. Velvet: algorithms for de novo short read assembly using de Bruijn graphs. Genome research. 2008;18(5):821–9. 10.1101/gr.074492.107 18349386 PMC2336801

[49] Schulz MH, Zerbino DR, Vingron M, Birney E. Oases: robust de novo RNA-seq assembly across the dynamic range of expression levels. Bioinformatics. 2012;28(8):1086–92. 10.1093/bioinformatics/bts094 22368243 PMC3324515

[50] Wiebe NJ, Meyer IM. TRANSAT—method for detecting the conserved helices of functional RNA structures, including transient, pseudo-knotted and alternative structures. PLoS Comput Biol. 2010;6(6):e1000823 Epub 2010/07/01. 10.1371/journal.pcbi.1000823 20589081 PMC2891591

[51] Lai D, Proctor JR, Zhu JY, Meyer IM. R-CHIE: a web server and R package for visualizing RNA secondary structures. Nucleic acids research. 2012;40(12):e95 Epub 2012/03/22. 10.1093/nar/gks241 22434875 PMC3384350

