# Supplementary figures and images for: Identification and Characterization of Two Novel RNA Viruses from Anopheles gambiae Species Complex Mosquitoes
Source: PLoS One. 2016 May 3;11(5):e0153881. doi: 10.1371/journal.pone.0153881 (PMC4854438; doi:10.1371/journal.pone.0153881)

A

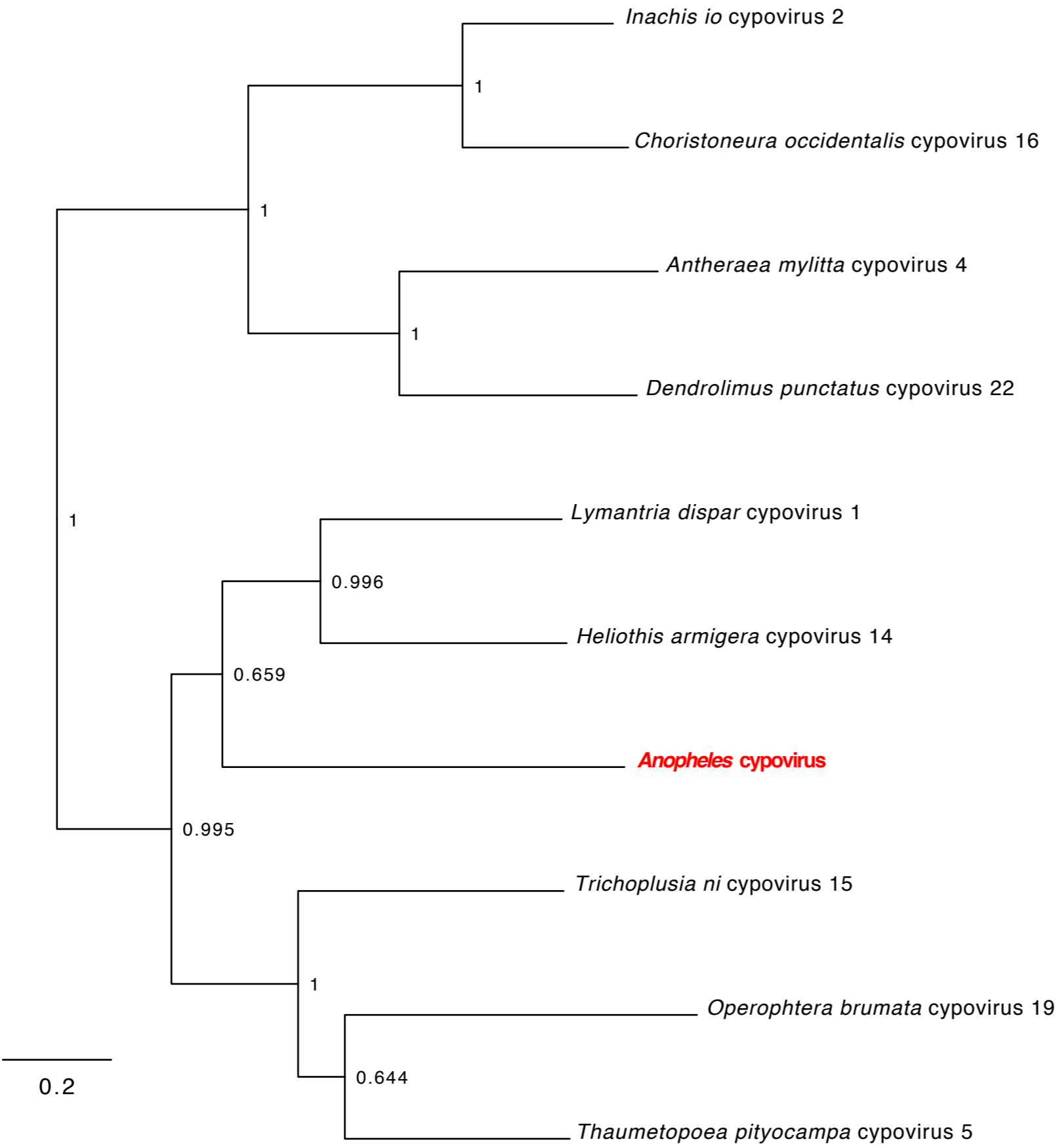

B

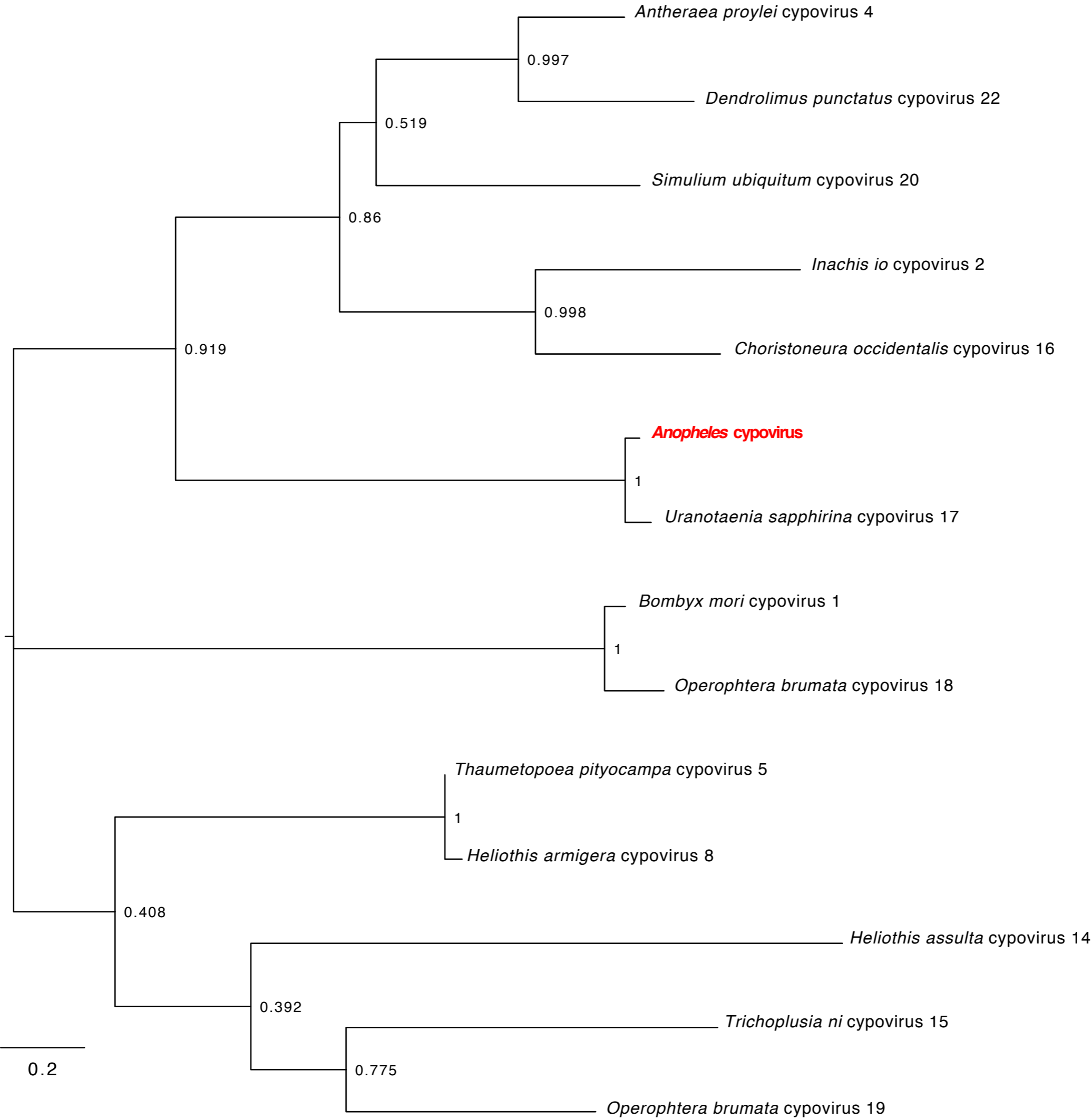

Supplement: S1 Fig — (A) Maximum likelihood tree of RNA-dependent RNA polymerase (RdRp) sequences of Cypoviruses, (B) Maximum likelihood tree of Polyhedrin sequences of Cypoviruses. Multiple protein alignment was done using the partial deletion option in MEGA. ML bootstrap values (above 0.75) are indicated above branches. (PDF) [file pone.0153881.s001.pdf]

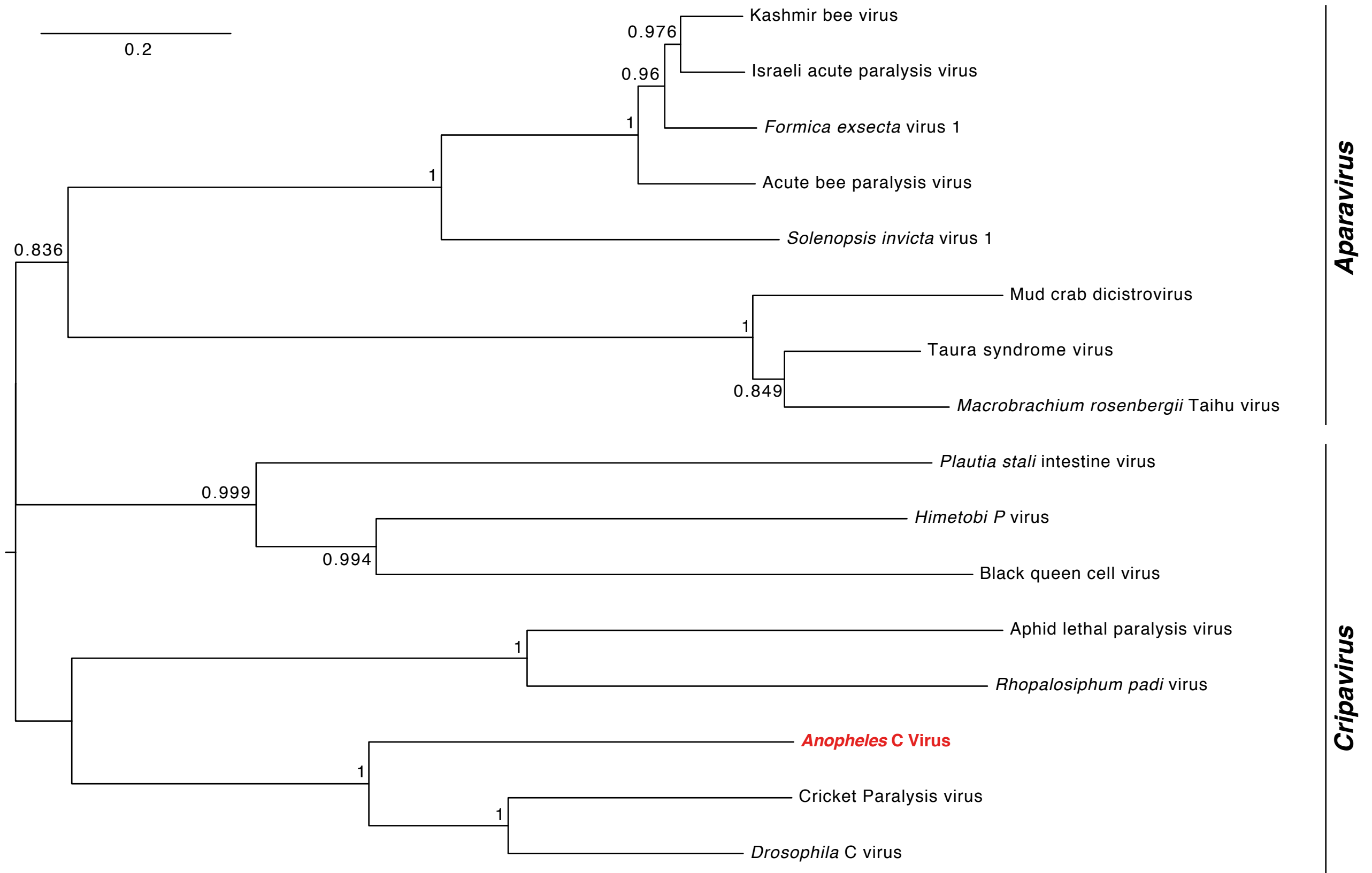

Supplement: S2 Fig — Neighbor-joining tree of non-structural polyprotein sequences of Dicistrovirus. Multiple protein alignment was done using the partial deletion option in MEGA. NJ bootstrap values above 0.75 are indicated above branches. The two genera of the Dicistroviridae family are indicated on the tree. (PDF) [file pone.0153881.s002.pdf]
