# Supplementary material for: Identification and Characterization of Two Novel RNA Viruses from Anopheles gambiae Species Complex Mosquitoes
Source: PLoS One. 2016 May 3;11(5):e0153881. doi: 10.1371/journal.pone.0153881 (PMC4854438; doi:10.1371/journal.pone.0153881)
Supplement: S3 Fig — F and R suffix represent respectively forward and reverse sequencing orientations. Sanger sequencing raw files are in S3 Zipped Archive. (PDF) [file pone.0153881.s003.pdf]

```
                                             10        20        30        40        50
 +2 CPV-PCRfragment.xdna             acgcgtggtttacctgaatcacaaattggaccatttacaggcggtatgaa
-24 Ng10CPV-R                                            ..............................
-28 Ng08CPV-R                                            ..............................
-16 W35R_Senegal                                         ..............................
-14 W32R_Cambodia                                        ..............................
-18 W35R_Senegal                                         ..............................
-12 W31R_Cambodia                                        ..............................
-20 W36R_Senegal                                         ..............................
+31 W44F_Cambodia                                                              ...........
+27 Ng08CPV-F                                                                  ...........
+11 W31F_Cambodia                                                               ..........
+17 W35F_Senegal                                                                ..........
+23 Ng10CPV-F                                                                           .
    CONSENSUS                        ACGCGTGGTTTACCTGAATCACAAATTGGACCATTTACAGGCGGTATGAA

                                             60        70        80        90       100
 +2 CPV-PCRfragment.xdna             gcgtcttctgcaactaagtgatgaacctggtggagcgtggaagcaggttg
-24 Ng10CPV-R                        ..................................................
-28 Ng08CPV-R                        ..................................................
-16 W34R_Senegal                     ..................................................
-14 W32F_Cambodia                    ..................................................
-18 W35R_Senegal                     ..................................................
-12 W31R_Cambodia                    ..................................................
-20 W36R_Senegal                     ..................................................
+31 W44F_Cambodia                    ..................................................
+27 Ng08CPV-F                        ..................................................
+11 W31F_Cambodia                    ..................................................
+17 W35F_Senegal                     ..................................................
+23 Ng10CPV-F                        ..................................................
+13 W32F_Cambodia                       ...............................................
+19 W36F_Senegal                            ...........................................
    CONSENSUS                        GCGTCTTCTGCAACTAAGTGATGAACCTGGTGGAGCGTGGAAGCAGGTTG

                                            110       120       130       140       150
 +2 CPV-PCRfragment.xdna             agatgtatgtatcaggtacacttgaggccgtggtattcgaaggtagtcat
-24 Ng10CPV-R                        ..................................................
-28 Ng08CPV-R                        ..................................................
-16 W34R_Senegal                     ..................................................
-14 W32R_Cambodia                    ..................................................
-18 W35R_Senegal                     ..................................................
-12 W31R_Cambodia                    ..................................................
-20 W36R_Senegal                     ..................................................
+31 W44F_Cambodia                    ..................................................
+27 Ng08CPV-F                        ..................................................
+11 W31F_Cambodia                    ..................................................
+17 W35F_Senegal                     ..................................................
+23 Ng10CPV-F                        ..................................................
+13 W32F_Cambodia                    ..................................................
+19 W36F_Senegal                     ..................................................
+15 W34F_Senegal                                    ...................................
    CONSENSUS                        AGATGTATGTATCAGGTACACTTGAGGCCGTGGTATTCGAAGGTAGTCAT

                                            160       170       180       190       200
 +2 CPV-PCRfragment.xdna             gcactaacatcaactacttacgagagtaagatttttggaagtgttaagaa
-24 Ng10CPV-R                        ..................................................
-28 Ng08CPV-R                        ..................................................
-16 W34R_Senegal                     ..................................................
-14 W32R_Cambodia                    ..................................................
-18 W35R_Senegal                     ..................................................
-12 W31R_Cambodia                    ..................................................
-20 W36R_Senegal                     ..................................................
+31 W44F_Cambodia                    ..................................................
+27 Ng08CPV-F                        ..................................................
+11 W31F_Cambodia                    ..................................................
+17 W35F_Senegal                     ..................................................
+23 Ng10CPV-F                        ..................................................
+13 W32F_Cambodia                    ..................................................
+19 W36F_Senegal                     ..................................................
+15 W34F_Senegal                     ..................................................
    CONSENSUS                        GCACTAACATCAACTACTTACGAGAGTAAGATTTTTGGAAGTGTTAAGAA
```

```
                                                    210       220       230       240       250
 +2 CPV-PCRfragment.xdna         ctatgcgggtacgtatccctcaggtagagctgacacgtcgtcacatcaca
-24 Ng10CPV-R                    ..................................................
-28 Ng08CPV-R                    ..................................................
-16 W34R_Senegal                 ..................................................
-14 W32R_Cambodia                ..................................................
-18 W35R_Senegal                 ..................................................
-12 W31R_Cambodia                ..................................................
-20 W36R_Senegal                 ..................................................
+31 W44F_Cambodia                ..................................................
+27 Ng08CPV-F                    ..................................................
+11 W31F_Cambodia                ..................................................
+17 W35F_Senegal                 ..................................................
+23 Ng10CPV-F                    ..................................................
+13 W32F_Cambodia                ..................................................
+19 W36F_Senegal                 ..................................................
+15 W34F_Senegal                 ..................................................
    CONSENSUS                    CTATGCGGGTACGTATCCCTCAGGTAGAGCTGACACGTCGTCACATCACA

                                                    260       270       280       290       300
 +2 CPV-PCRfragment.xdna         ctaaggttttggaaggtgcgacccgtggtaatgaaatgcgaagaagaacg
-24 Ng10CPV-R                    ..................................................
-28 Ng08CPV-R                    ..................................................
-16 W34R_Senegal                 ..................................................
-14 W32R_Cambodia                ..................................................
-18 W35R_Senegal                 ..................................................
-12 W31R_Cambodia                ..................................................
-20 W36R_Senegal                 ..................................................
+31 W44F_Cambodia                ..................................................
+27 Ng08CPV-F                    ..................................................
+11 W31F_Cambodia                ..................................................
+17 W35F_Senegal                 ..................................................
+23 Ng10CPV-F                    ..................................................
+13 W32F_Cambodia                ..................................................
+19 W36F_Senegal                 ..................................................
+15 W34F_Senegal                 ..................................................
    CONSENSUS                    CTAAGGTTTTGGAAGGTGCGACCCGTGGTAATGAAATGCGAAGAAGAACG

                                                    310       320       330       340       350
 +2 CPV-PCRfragment.xdna         gatgagcgaatcgtgcaccatgcgtcgacgattgtaatgtcacgtaatat
-24 Ng10CPV-R                    ..T...............................................
-28 Ng08CPV-R                    ..T...............................................
-16 W34R_Senegal                 ..................................................
-14 W32R_Cambodia                ..................................................
-18 W35R_Senegal                 ..................................................
-12 W31R_Cambodia                ..................................................
-20 W36R_Senegal                 ..................................................
+31 W44F_Cambodia                ..T...............................................
+27 Ng08CPV-F                    ..T...............................................
+11 W31F_Cambodia                ..................................................
+17 W35F_Senegal                 ..................................................
+23 Ng10CPV-F                    ..T...............................................
+13 W32F_Cambodia                ..................................................
+19 W36F_Senegal                 ..................................................
+15 W34F_Senegal                 ..................................................
    CONSENSUS                    GACGAGCGAATCGTGCACCATGCGTCGACGATTGTAATGTCACGTAATAT
```

```
                                         360       370       380       390       400
 +2 CPV-PCRfragment.xdna          gggtgatgataagtcagacgtatacacaggatcatttccaaatgttatat
-24 Ng10CPV-R                     ..................................................
-28 Ng08CPV-R                     .....................R............................
-16 W34R_Senegal                  ..................................................
-14 W32R_Cambodia                 ..................................................
-18 W35R_Senegal                  ..................................................
-12 W31R_Cambodia                 ..................................................
-20 W36R_Senegal                  ..................
+31 W44F_Cambodia                 .....................R............................
+27 Ng08CPV-F                     .....................R............................
+11 W31F_Cambodia                 ..................................................
+17 W35F_Senegal                  ..................................................
+23 Ng10CPV-F                     ..................................................
+13 W32F_Cambodia                 ..................................................
+19 W36F_Senegal                  ..................................................
+15 W34F_Senegal                  ..................................................
    CONSENSUS                     GGGTGATGATAAGTCAGACGTATACACAGGATCATTTCCAAATGTTATAT

                                         410       420       430       440       450
 +2 CPV-PCRfragment.xdna          cacaattagtgagcgataaagatgtactcgcacaattgggttttaagaca
-24 Ng10CPV-R                     ..................................................
-28 Ng08CPV-R                     ..................................................
-16 W34R_Senegal                  ........
-14 W32R_Cambodia                 ..................................................
-18 W35R_Senegal                  ..................................................
-12 W31R_Cambodia                 ..................................................
+31 W44F_Cambodia                 ..................................................
+27 Ng08CPV-F                     ..................................................
+11 W31F_Cambodia                 ..................................................
+17 W35F_Senegal                  ..................................................
+23 Ng10CPV-F                     ..................................................
+13 W32F_Cambodia                 ..................................................
+19 W36F_Senegal                  ..................................................
+15 W34F_Senegal                  ..................................................
    CONSENSUS                     CACAATTAGTGAGCGATAAAGATGTACTCGCACAATTGGGTTTTAAGACA

                                         460       470       480       490       500
 +2 CPV-PCRfragment.xdna          gatgcagatctctcgagtcacaacggagaattcttgcaacaacatgtatg
-24 Ng10CPV-R                     .......................C.....
-28 Ng08CPV-R                     .......................C.....
-14 W32R_Cambodia                 .........................
-18 W35R_Senegal                  .........................
-12 W31R_Cambodia                 .........................
+31 W44F_Cambodia                 .......................C..........................
+27 Ng08CPV-F                     .......................C..........................
+11 W31F_Cambodia                 ..................................................
+17 W35F_Senegal                  ..................................................
+23 Ng10CPV-F                     .......................C..........................
+13 W32F_Cambodia                 ..................................................
+19 W36F_Senegal                  ..................................................
+15 W34F_Senegal                  ..................................................
    CONSENSUS                     GATGCAGATCTCTCGAGTCACAATGGAGAATTCTTGCAACAACATGTATG

                                         510       520
 +2 CPV-PCRfragment.xdna          tagaggaaggctagtcggaaaacc
+31 W44F_Cambodia                 ....
+27 Ng08CPV-F                     ....
+11 W31F_Cambodia                 ....
+17 W35F_Senegal                  ....
+23 Ng10CPV-F                     ....
+13 W32F_Cambodia                 ....
+19 W36F_Senegal                  ....
+15 W34F_Senegal                  ....
    CONSENSUS                     TAGAGGAAGGCTAGTCGGAAAACC
```
