# Supplementary material for: Identification and Characterization of Two Novel RNA Viruses from Anopheles gambiae Species Complex Mosquitoes
Source: PLoS One. 2016 May 3;11(5):e0153881. doi: 10.1371/journal.pone.0153881 (PMC4854438; doi:10.1371/journal.pone.0153881)
Supplement: S4 Fig — F and R suffix represent respectively forward and reverse sequencing orientations. Sanger sequencing raw files are in S3 Zipped Archive. (PDF) [file pone.0153881.s004.pdf]

```
                                             10        20        30        40        50
 +1 ACV-PCRfragment.xdna            caaggaggctttttgagtgctcctgcgaaagctgtaacgataggacaaga
-32 W47R_Senegal                                        ..............................
-21 Ng10ACV-R                                           ..............................
-25 Ng08ACV-R                                           ..............................
 -8 An.dirus4-R                                         ..............................
 -6 An.dirus3-R                                         ..............................
 +5 An.dirus3-F                                                         ..............
 +7 An.dirus4-F                                                         ..............
+26 Ng08ACV-F                                                                      ...
    CONSENSUS                       CAAGGAGGCTTTTTGAGTGCTCCTGCGAAAGCTGTAACGATAGGACAAGA

                                            60        70        80        90       100
 +1 ACV-PCRfragment.xdna            tttgaacgcgaacctagataaggtttgctcatttcttgaggatgctttac
-32 W47R_Senegal                    ...........................Y......................
-21 Ng10ACV-R                       ...........................Y......................
-25 Ng08ACV-R                       ...........................T......................
 -8 An.dirus4-R                     ..................................................
 -6 An.dirus3-R                     ..................................................
 +5 An.dirus3-F                     ..................................................
 +7 An.dirus4-F                     ..................................................
+26 Ng08ACV-F                       ...........................T......................
+33 W47F_Senegal                         ......................Y......................
+22 Ng10ACV-F                                        ..........Y......................
    CONSENSUS                       TTTGAACGCGAACCTAGATAAGGTTTGCTCATTTCTTGAGGATGCTTTAC

                                           110       120       130       140       150
 +1 ACV-PCRfragment.xdna            caggtatggtcgagcacgttacgctcgtagcacaaaatacatccgcgtca
-32 W47R_Senegal                    ..................................................
-21 Ng10ACV-R                       ..................................................
-25 Ng08ACV-R                       ..................................................
 -8 An.dirus4-R                     ..................................................
 -6 An.dirus3-R                     ..................................................
 +5 An.dirus3-F                     ..................................................
 +7 An.dirus4-F                     ..................................................
+26 Ng08ACV-F                       ..................................................
+33 W47F_Senegal                    ..................................................
+22 Ng10ACV-F                       ..................................................
    CONSENSUS                       CAGGTATGGTCGAGCACGTTACGCTCGTAGCACAAAATACATCCGCGTCA

                                           160       170       180       190       200
 +1 ACV-PCRfragment.xdna            gccaaggtgttatctgacgagttgatcaaatcaatgctttgcattgtttt
-32 W47R_Senegal                    ..................................................
-21 Ng10ACV-R                       ..................................................
-25 Ng08ACV-R                       ..................................................
 -8 An.dirus4-R                     ..................................................
 -6 An.dirus3-R                     ..................................................
 +5 An.dirus3-F                     ..................................................
 +7 An.dirus4-F                     ..................................................
+26 Ng08ACV-F                       ..................................................
+33 W47F_Senegal                    ..................................................
+22 Ng10ACV-F                       ..................................................
    CONSENSUS                       GCCAAGGTGTTATCTGACGAGTTGATCAAATCAATGCTTTGCATTGTTTT

                                           210       220       230       240       250
 +1 ACV-PCRfragment.xdna            gatttgcttgttgattgaaaccaagttctataagaccgctttcgcggtac
-32 W47R_Senegal                    ..................................................
-21 Ng10ACV-R                       ..................................................
-25 Ng08ACV-R                       ..................................................
 -8 An.dirus4-R                     ..................................................
 -6 An.dirus3-R                     ..................................................
 +5 An.dirus3-F                     ..................................................
 +7 An.dirus4-F                     ..................................................
+26 Ng08ACV-F                       ..................................................
+33 W47F_Senegal                    ..................................................
+22 Ng10ACV-F                       ..................................................
    CONSENSUS                       GATTTGCTTGTTGATTGAAACCAAGTTCTATAAGACCGCTTTCGCGGTAC
```

```
                                            260       270       280       290       300
 +1 ACV-PCRfragment.xdna           ttatagtggttgctctacgtgttttcgggtacagtgagcaaataattgag
-32 W47R_Senegal                   ..................................................
-21 Ng10ACV-R                      ..................................................
-25 Ng08ACV-R                      ..................................................
 -8 An.dirus4-R                    ..................................................
 -6 An.dirus3-R                    ..................................................
 +5 An.dirus3-F                    ..................................................
 +7 An.dirus4-F                    ..................................................
+26 Ng08ACV-F                      ..................................................
+33 W47F_Senegal                   ..................................................
+22 Ng10ACV-F                      ..................................................
    CONSENSUS                      TTATAGTGGTTGCTCTACGTGTTTTCGGGTACAGTGAGCAAATAATTGAG

                                            310       320       330       340       350
 +1 ACV-PCRfragment.xdna           acagctatggacatgtatcgcgtaattagggctccaaaggctcaaggtaa
-32 W47R_Senegal                   ..................................................
-21 Ng10ACV-R                      ..................................................
-25 Ng08ACV-R                      ..................................................
 -8 An.dirus4-R                    ..................................................
 -6 An.dirus3-R                    ..................................................
 +5 An.dirus3-F                    ..................................................
 +7 An.dirus4-F                    ..................................................
+26 Ng08ACV-F                      ..................................................
+33 W47F_Senegal                   ..................................................
+22 Ng10ACV-F                      ..................................................
    CONSENSUS                      ACAGCTATGGACATGTATCGCGTAATTAGGGCTCCAAAGGCTCAAGGTAA

                                            360       370       380       390       400
 +1 ACV-PCRfragment.xdna           tatggaagatgtcgttttccatccgtggttgaacacgtgtggaaagttga
-32 W47R_Senegal                   ..................................................
-21 Ng10ACV-R                      ..................................................
-25 Ng08ACV-R                      ..................................................
 -8 An.dirus4-R                    ..................................................
 -6 An.dirus3-R                    ..................................................
 +5 An.dirus3-F                    ..................................................
 +7 An.dirus4-F                    ..................................................
+26 Ng08ACV-F                      ..................................................
+33 W47F_Senegal                   ..................................................
+22 Ng10ACV-F                      ..................................................
    CONSENSUS                      TATGGAAGATGTCGTTTTCCATCCGTGGTTGAACACGTGTGGAAAGTTGA

                                            410       420       430       440       450
 +1 ACV-PCRfragment.xdna           ttttcctacttatcgctgtcctgtgtctcaagaaattaccaggaaagaac
-32 W47R_Senegal                   ..................................................
-21 Ng10ACV-R                      ..........................
-25 Ng08ACV-R                      ..................................................
 -8 An.dirus4-R                    ..................................................
 -6 An.dirus3-R                    ..................................................
 +5 An.dirus3-F                    ..................................................
 +7 An.dirus4-F                    ..................................................
+26 Ng08ACV-F                      ..................................................
+33 W47F_Senegal                   ..................................................
+22 Ng10ACV-F                      ..................................................
    CONSENSUS                      TTTTCCTACTTATCGCTGTCCTGTGTCTCAAGAAATTACCAGGAAAGAAC

                                            460       470       480       490
 +1 ACV-PCRfragment.xdna           gacgtagacactttcatgcgcaggctcgacagcttacccaaagc
-32 W47R_Senegal                   ..
 -8 An.dirus4-R                    ...........
 -6 An.dirus3-R                    ...........
 +5 An.dirus3-F                    ........................
 +7 An.dirus4-F                    ........................
+26 Ng08ACV-F                      ........................
+33 W47F_Senegal                   ........................
+22 Ng10ACV-F                      ........................
    CONSENSUS                      GACGTAGACACTTTCATGCGCAGGCTCGACAGCTTACCCAAAGC
```
