# Supplementary material for: Identification and Characterization of Two Novel RNA Viruses from Anopheles gambiae Species Complex Mosquitoes
Source: PLoS One. 2016 May 3;11(5):e0153881. doi: 10.1371/journal.pone.0153881 (PMC4854438; doi:10.1371/journal.pone.0153881)
Supplement: S2 Table — Final suffix indicates forward, F, or reverse, R, sense of primers. (DOCX) [file pone.0153881.s012.docx]

| AnCPVs1-F | ACGCGTGGTTTACCTGAATC |
| --- | --- |
| AnCPVs1-R | GGTTTTCCGACTAGCCTTCC |
| AnCPVs10-F | AATGGCCGATCTATCACTGG |
| AnCPVs10-R | TTCGATCACATTACCGAGCA |
| AnCV-F | CAAGGAGGCTTTTTGAGTGC |
| AnCV-R | GCTTTGGGTAAGCTGTCGAG |
| S7-F | AGGCGATCATCATCTACG |
| S7-R | GTAGCTGCTGCAAACTTC |

**Table S11.** Primers used for one step RT-PCR. Final suffix indicates forward, F, or reverse, R, sense of primers.
